# Microglia and monocytes synergistically promote the transition from acute to chronic pain after nerve injury

**DOI:** 10.1038/ncomms12029

**Published:** 2016-06-28

**Authors:** Jiyun Peng, Nan Gu, Lijun Zhou, Ukpong B Eyo, Madhuvika Murugan, Wen-Biao Gan, Long-Jun Wu

**Affiliations:** 1 Department of Cell Biology and Neuroscience, Rutgers University, Piscataway, New Jersey 08854, USA; 2 Department of Anesthesia, Xijing Hospital, Fourth Military Medical University, Xi'an 710032, China; 3 Department of Physiology and Pain Research Center, Zhongshan School of Medicine, Sun Yet-Sen University, Guangzhou 510080, China; 4 Skirball Institute, Department of Neuroscience and Physiology, New York University School of Medicine, New York, New York 10016, USA

## Abstract

Microglia and peripheral monocytes contribute to hypersensitivity in rodent models of neuropathic pain. However, the precise respective function of microglia and peripheral monocytes has not been investigated in these models. To address this question, here we combined transgenic mice and pharmacological tools to specifically and temporally control the depletion of microglia and monocytes in a mouse model of spinal nerve transection (SNT). We found that although microglia and monocytes are required during the initiation of mechanical allodynia or thermal hyperalgesia, these cells may not be as important for the maintenance of hypersensitivity. Moreover, we demonstrated that either resident microglia or peripheral monocytes are sufficient in gating neuropathic pain after SNT. We propose that resident microglia and peripheral monocytes act synergistically to initiate hypersensitivity and promote the transition from acute to chronic pain after peripheral nerve injury.

## Abstract (web summary)

Microglia and monocytes contribute to neuropathic pain states, but the precise role of the two cell types is not clear. Here Peng *et al.* use temporally controlled ablation of monocytes and microglia in mice to show that these cells work together to initiate neuropathic-pain like behaviour, but are less important in the maintenance phase.

## Graphical abstract

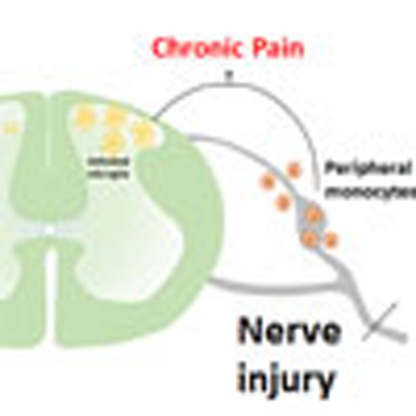

Neuropathic pain is a chronic pain state resulting from peripheral or central nerve injury due to trauma (for example, amputation and nerve injury) or systemic disease (for example, diabetes, viral infection and cancer)^1^,^2^. A key event in neuropathic pain is the transition from acute to chronic pain; however, the underlying mechanisms of this transition remain largely unknown. Our understanding of neuropathic pain mechanisms has expanded from being focused largely on neurocentric mechanisms to include neuro-glial interactions^3^,^4^,^5^. Spinal glia including astrocytes and microglia become activated after peripheral nerve injury, subsequently contributing to chronic pain by releasing a number of glial mediators that sensitize spinal neurons^6^,^7^,^8^,^9^. In addition, peripheral nerve injury recruits circulating monocytes to the injury sites, which can also release proinflammatory mediators that cause neuronal hyperactivity in the periphery^10^. These studies raise the intriguing possibility that central resident microglia and peripheral monocytes may control the transition from the acute to chronic pain.

Microglia comprise a unique subset of glial cells (5–10%) as the principal immune cells in the central nervous system (CNS). Resting microglia have highly dynamic processes by which they survey the microenvironment in the brain and spinal cord^11^,^12^,^13^,^14^,^15^. After peripheral nerve injury, spinal microglia transform from resting to reactive states, exhibiting marked changes in cell surface protein expression, and releasing a variety of proinflammatory mediators^7^,^16^,^17^,^18^,^19^. Inhibiting these microglia-derived molecules strongly suppresses pain-like hypersensitivity, suggesting that microglial response is a critical component of neuropathic pain. However, no evidence directly addresses the exact role of microglia in the initiation or maintenance of neuropathic pain.

Peripheral nerve injury is also associated with the recruitment of peripheral monocytes and their infiltration into the CNS parenchyma. Indeed, it was demonstrated that blood-borne circulating monocytes infiltrate into the spinal cord and play an essential role in neuropathic pain development^20^. However, the role of peripheral monocytes in neuropathic pain is still debated^21^,^22^. Nevertheless, the importance of peripheral monocytes in pain-like hypersensitivity is evident from the studies showing dissociation between activation of spinal microglia and chronic pain in certain animal models of peripheral nerve injury^23^,^24^ and in chemotherapy-induced pain^25^. So far, no report has pinpointed the specific role of resident microglia and peripheral monocytes in neuropathic hypersensitivity.

Using transgenic mice that enabled us to ablate resident microglia and peripheral monocytes in a temporally controlled fashion, we delineated the time window during which microglia and monocytes are required for the development of neuropathic pain in a mouse model of spinal nerve transection (SNT). In addition, we combined pharmacological and genetic tools to ablate resident microglia and peripheral monocytes, and determined their respective functions in neuropathic hypersensitivity. Our results indicate that depletion of both resident microglia and peripheral monocytes completely prevented the development of neuropathic pain. However, either resident microglia or peripheral macrophages are critical for the initiation of neuropathic pain, suggesting that they act synergistically to promote the transition from acute to chronic pain after peripheral nerve injury.

## Results

### CX_3_CR1^+^ cell depletion prevents hypersensitivity after SNT

**Figure 1:**
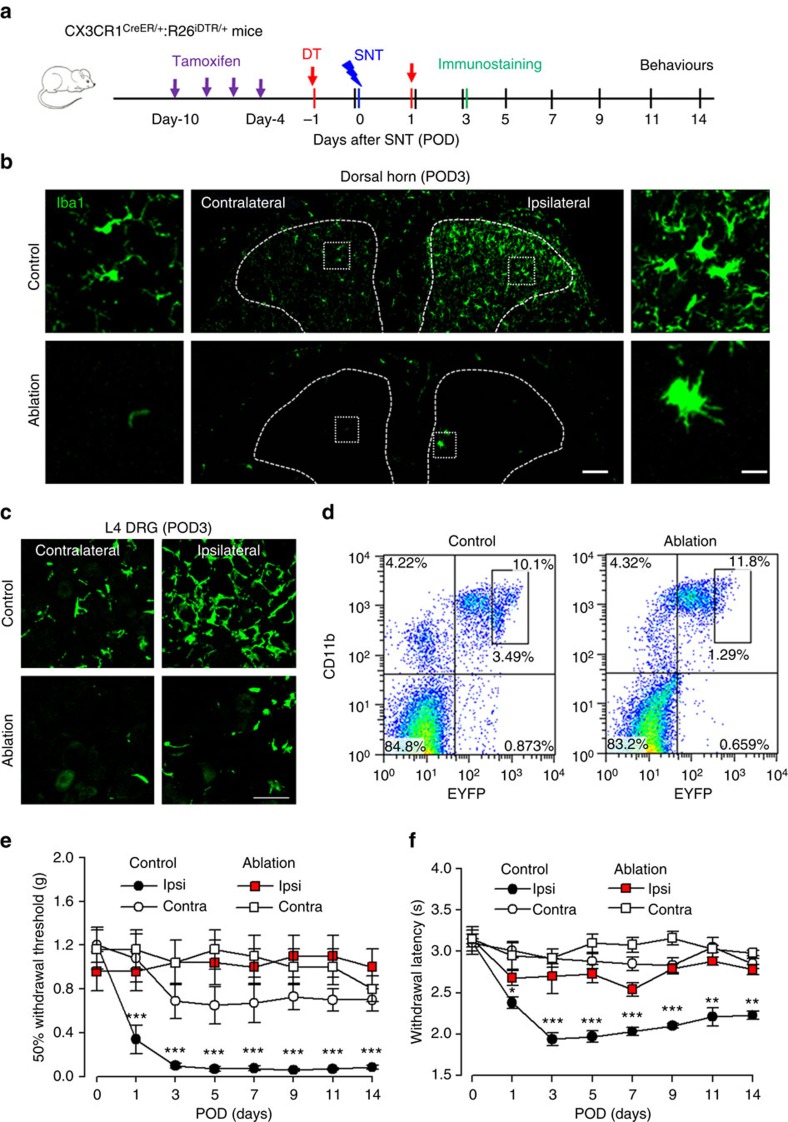
Ablation of CX_3_CR1^+^ cell population prevents neuropathic pain development. (**a**) An experimental diagram showing the timeline of drug treatments, SNT surgery, immunostaining and behavioural tests. (**b**) Confocal images showing Iba1-positive microglia in the dorsal horn of CX_3_CR1^CreER/+^:R26^iDTR/+^ mice on the ipsilateral and contralateral side at POD3 after SNT in control and ablation mice. Scale bar, 100 μm. Left and right: representative higher-magnification images from boxed regions showing microglial morphology. Scale bar, 20 μm. (**c**) Representative images of Iba-1-stained macrophages from both contralateral and ipsilateral L4 DRG in ablation and control group. Scale bar, 50 μm. (**d**) Flow cytometry signatures of blood monocytes depicting CD11b^+^CX_3_CR1^+^ population in ablation and control groups. The CX_3_CR1 expression level was indicated by intensity of EYFP fluorescence (*n*=3 for each group). (**e**,**f**) Behavioural assays showing mechanical allodynia (**e**) and thermal hyperalgesia (**f**) in ablation and control groups. (Data represent mean±s.e.m., *n*=9 for ablation group and 7 for control groups. ***P<0.001, **P<0.01, ablation ipsi versus control ipsi for both mechanical, *U*-test, and thermal response, *t*-test.) CX_3_CR1^CreER/+^:R26^iDTR/+^ mice with DT only were considered to be controls and mice with both TM+DT treatment was ablation group.

The chemokine receptor CX_3_CR1 is predominantly expressed by microglia in the CNS, but is also found in a subset of monocytes, macrophages, natural killer cells and dendritic cells in the periphery^26^. To study the role of CX_3_CR1^+^ cells in neuropathic pain, we used a strategy to temporally and specifically express the diphtheria toxin receptor (DTR) in CX_3_CR1^+^ cells by crossing CX_3_CR1^CreER^ mice with Rosa26-stop-DTR mice (CX_3_CR1^CreER/+^:R26^iDTR/+^) and subsequent induction of cre recombinase by tamoxifen (TM) injection^27^. Therefore, we were able to control the ablation of CX_3_CR1^+^ cells by diphtheria toxin (DT) application and directly investigated the temporal role of CX_3_CR1^+^ cells in chronic pain behaviours after L4 SNT, a well-established mouse model of neuropathic pain. TM (150 mg kg^−1^ in corn oil, 4 doses with 2-day intervals) or corn oil control was intraperitoneally (i.p.) injected to CX_3_CR1^CreER/+^:R26^iDTR/+^ mice before SNT and DT (50 μg kg^−1^, i.p., 3 days after last TM dose) was administered 1 day before and 1 day after SNT to deplete CX_3_CR1^+^ cells (Fig. 1a). At postoperative day 3 (POD3), we examined the CNS microglia and dorsal root ganglia (DRG) macrophages by immunostaining for Iba1, as well as blood monocytes that express CX_3_CR1 by flow cytometry (Fig. 1b–d). CD11b, another marker for microglia and macrophages, was also used to confirm the ablation efficiency in spinal dorsal horn (Supplementary Fig. 1a). In CX_3_CR1^CreER/+^:R26^iDTR/+^ mice without TM-induced DTR expression (control), SNT markedly increased the number of microglia in the ipsilateral dorsal horn and resident macrophages in DRGs compared with contralateral sides at POD3. However, in CX_3_CR1^CreER/+^:R26^iDTR/+^ mice with both TM and DT injection (ablation), spinal microglia and DRG macrophages in both contralateral and ipsilateral sides were largely depleted (Fig. 1b,c and Supplementary Fig. 1a,c). In these CX_3_CR1^CreER/+^ mice in which the CreER-encoding gene was followed by an IRES-EYFP element^27^, a subset of CD11b^+^/EYFP^++^-positive blood cells that are monocytes with high CX_3_CR1 expression was also depleted (Fig. 1d and Supplementary Fig. 1d). In addition, microglia were depleted in most supraspinal brain regions, such as the rostral ventromedial medulla, anterior cingulate cortex and hippocampus (Supplementary Fig. 1b,c). Together, these results indicate that our ablation strategy was able to successfully deplete CX_3_CR1^+^ cells, including microglia in the brain and spinal cord, DRG macrophages and CX_3_CR1^+^ monocytes.

Next, we wanted to know whether depletion of CX_3_CR1^+^ cells affected mouse pain behaviours. First, we measured acute pain behaviours in mice with CX_3_CR1^+^ cell depletion. We found that CX_3_CR1^+^ cell depletion did not alter acute pain responses to either mechanical or thermal stimulation (POD0, Fig. 1e,f). Also, there was no difference in tail flick tests between control and ablation groups (Supplementary Fig. 2a). Motor coordination in the rotarod test was similar between the two groups, although inter-session motor learning was impaired in mice with CX_3_CR1^+^ cell depletion (Supplementary Fig. 2b). Second, we compared chronic pain behaviours after peripheral nerve injury in mice with or without CX_3_CR1^+^ cell depletion, using the SNT mouse model of neuropathic pain. We found that mechanical and thermal hypersensitivity following SNT were completely abolished in mice with CX_3_CR1^+^ cell depletion compared with control mice. Both mechanical allodynia and thermal hyperalgesia were prevented after SNT by such ablation and lasted at least 2 weeks (Fig. 1e,f). A recent report showed a sex difference in the role of microglia in mechanical allodynia in male and female mice 7 days after nerve injury^28^. Interestingly, we found that mechanical and thermal hypersensitivity following SNT were completely abolished in both male and female mice with CX_3_CR1^+^ cell-depleted at POD3 compared with control mice (Supplementary Fig. 3). These results suggest that CX_3_CR1^+^ cells equally participated in the neuropathic pain development in both male and female mice.

The CX_3_CR1^+^ cell depletion strategy using DT in CX_3_CR1^CreER/+^:R26^iDTR/+^ mice induces microglial cell death and may cause local inflammatory environment change. Thus, we introduced a second microglia- and macrophage-manipulating method to confirm the role of CX_3_CR1^+^ cell population in neuropathic pain (Supplementary Fig. 4). Colony-stimulating factor 1 receptor (CSF1R), a key regulator of myeloid lineage cells, is required for microglia survival in the adult brain, and CSF1R inhibitor PLX3397 is able to deplete microglia in the brain^29^. Since PLX3397 is not commercially available, here we blocked the CSF-1 pathway by neutralizing CSF-1 with antibody (200 ng in 5 μl ACSF) through daily intrathecal injections from POD0 to POD5 after SNT surgery (Supplementary Fig. 4a). We found that neutralizing CSF-1 antibody was not able to completely ablate microglia and macrophages, but it suppressed microglia and macrophage numbers by reducing their proliferation, which is consistent with a recent study showing that CSF-1 signalling is critical for microglial activation after peripheral nerve injury^30^. The proliferation of spinal microglia and DRG macrophages was examined by a proliferating marker Ki-67 staining at POD3 after SNT. Compared with vehicle-treated mice (CX_3_CR1^GFP/+^ mice), the number of Ki-67^+^ proliferating dorsal horn microglia or DRG macrophages were markedly reduced in CSF-1 antibody-treated mice (Supplementary Fig. 4b,c). Consistently, we found that although the vehicle-treated mice developed chronic neuropathic pain normally, both mechanical allodynia and thermal hyperalgesia were significantly reversed in the CSF-1-neutralizing antibody-treated mice (Supplementary Fig. 4d,e). These results confirmed that manipulation of the number of spinal microglia and DRG macrophages is able to affect neuropathic pain development and replicate some aspects of CX_3_CR1^+^ cell depletion strategy using CX_3_CR1^CreER/+^:R26^iDTR/+^ mice.

### Repopulated CX3CR1^+^ cells cannot recover hypersensitivity

**Figure 2:**
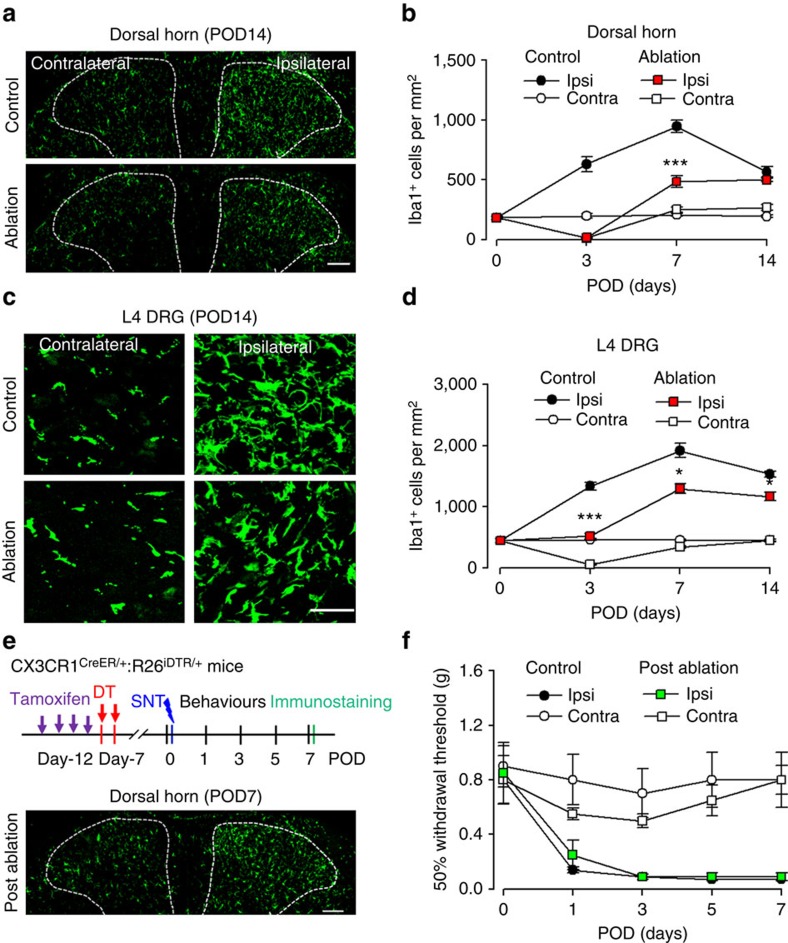
Repopulated microglia and macrophages are able to engage in the development of neuropathic pain. (**a**) Representative images showing repopulation of Iba1-positive spinal microglia at POD14 in control and pre-SNT ablation mice. Scale bar, 100 μm. (**b**) Graphical representation showing the time course of ablation and repopulation of dorsal horn microglia in both ipsilateral and contralateral sides after SNT in control and ablation groups (*n*=3 mice for each group, *n*=3 images for each animal, ***P<0.001, ablation ipsi versus control ipsi, *t*-test). (**c**) Representative images showing macrophage repopulation in the contralateral and ipsilateral DRGs at POD14. Scale bar, 50 μm. (**d**) Time course of macrophage repopulation in the contralateral and ipsilateral DRG after SNT in control and ablation groups (*n*=3 mice for each group, *n*=3 images for each animal, *P<0.05, ***P<0.001, ablation ipsi versus control ipsi, *t*-test). (**e**) An experimental diagram showing the timeline of experiments (upper) and a representative image showing the repopulated microglia in the spinal dorsal horn at POD7 after SNT (lower). Scale bar, 100 μm. (**f**) Mechanical allodynia after SNT in post-ablation mice when microglia and macrophages were repopulated. Data represent average 50% paw withdrawal threshold±s.e.m. (*n*=6 for each group; *P*>0.05 for all testing points, post-ablation ipsi versus control ipsi, *U*-test). CX_3_CR1^CreER/+^:R26^iDTR/+^ mice with DT only were considered to be controls and mice with both TM+DT treatment was ablation group.

A unique property of microglia and macrophages is their capacity to quickly repopulate the CNS after ablation^29^,^31^. Here we allowed the repopulation of spinal microglia and DRG macrophages and examined their function in neuropathic pain using CX_3_CR1^CreER/+^:R26^iDTR/+^ mice after SNT. We found that microglia in the spinal dorsal horn repopulated rapidly after their complete depletion at POD3. In both contralateral and ipsilateral dorsal horn, spinal microglia repopulated quickly at POD7 and POD14 in CX_3_CR1 cell-ablated mice (Fig. 2a,b). The morphology of the newly repopulated spinal microglia differed from that in age-matched control mice, exhibiting shorter processes and fewer branches at POD7 (Supplementary Fig. 5). In addition, macrophages in the injured L4 DRG also showed rapid repopulation at POD7 and POD14 (Fig. 2c,d). These results indicate that both spinal microglia and DRG macrophages undergo rapid repopulation after their depletion by DT in CX_3_CR1^CreER/+^:R26^iDTR/+^ mice. Although microglia and macrophages repopulated in the spinal cord and DRG within a week, the reduction in pain-like behaviours in CX_3_CR1 cell ablation mice was long-lasting (Fig. 1e,f). Therefore, these results suggest that repopulated microglia and macrophages after SNT are unable to re-establish neuropathic hypersensitivity.

To test whether repopulated microglia and macrophages are functional in the development of neuropathic pain, we depleted CX_3_CR1^+^ cells and allowed a week for their repopulation. Then SNT was performed in these mice (Fig. 2e). We confirmed that the number of repopulated spinal microglia and DRG macrophages in CX_3_CR1 cell-ablated mice was comparable to that in control mice (Fig. 2e). In these mice with repopulated microglia and macrophages (post ablation), we performed SNT and then neuropathic pain behaviours were tested. We found that mechanical allodynia developed normally in these mice and there was no difference in hypersensitivity between control and post-ablation groups (Fig. 2f). These results suggest that repopulated microglia and macrophages are functional being able to initiate neuropathic pain after peripheral nerve injury. Thus, we demonstrate that repopulated microglia and macrophages are able to engage in the development of neuropathic pain after *de novo* but not pre-existent nerve injury. These results suggest a critical period when signals derived from peripheral nerve injury, such as CSF-1 or ATP^30^,^32^, are able to recruit microglia to develop neuropathic pain-like hypersensitivity.

### Early ablation of CX_3_CR1^+^ cells reverses hypersensitivity

Depletion of CX_3_CR1^+^ cells abolished neuropathic pain behaviours after peripheral nerve injury (Fig. 1). However, the precise role of these cells at different stages of neuropathic pain development is still unknown. Taking advantage of CX_3_CR1^CreER/+^:R26^iDTR/+^ mice, we were able to temporally ablate CX_3_CR1^+^ cells by DT injection in the early (POD3–5) and late (at POD7–9) stages after SNT. Neuropathic hypersensitivity after SNT gradually developed from POD1–3 and was fully established after POD7. Consistently, the number of microglia in the ipsilateral spinal cord and L4 DRG increased during neuropathic pain development and peaked at POD7 (Fig. 2a–d).

**Figure 3:**
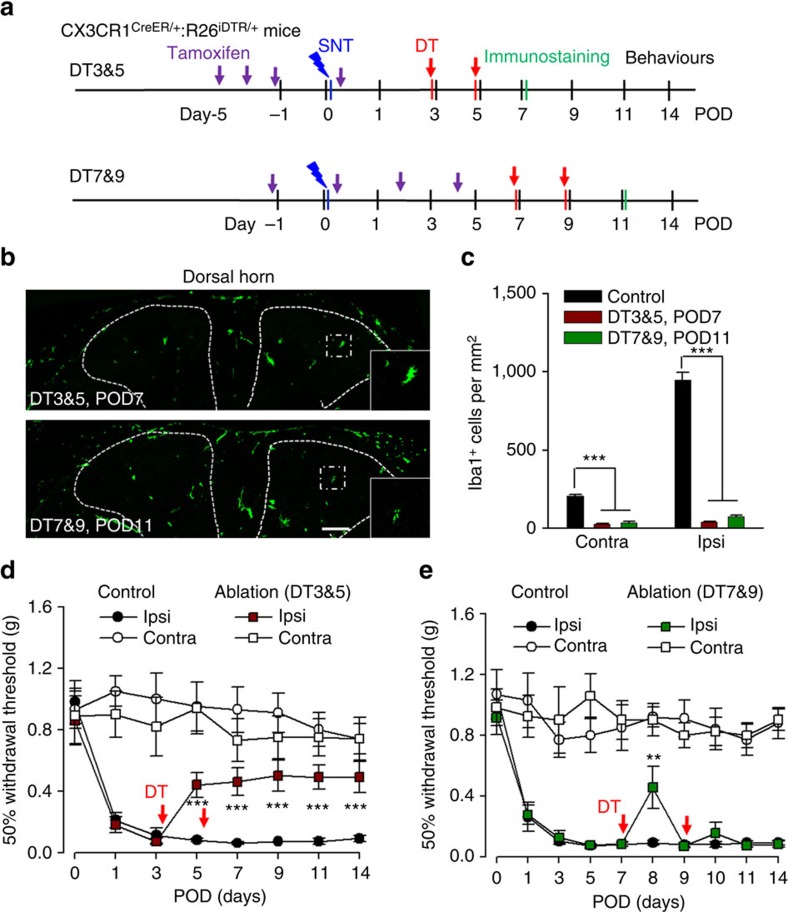
Early but not late CX_3_CR1^+^ cell ablation reverses neuropathic pain. (**a**) An experimental design showing the timeline for drug treatments, SNT surgery, immunostaining and behavioural assays to identify the critical window for microglia in SNT-induced neuropathic pain. For early phase: DT3&5, DT was applied at POD3 and 5. For late phase: DT7&9, DT was applied at POD7 and 9. (**b**) Confocal images of Iba1 staining showing microglial ablation in dorsal horn at POD7 in DT3&5 group and at POD11 in DT7&9 group. Representative higher-magnification images from boxed regions in larger images showing microglial morphology. Scale bar, 100 μm. (**c**) Pooled results showing the effect of ablation in the spinal dorsal horn (*n*=3 mice for each group, *n*=3 images for each animal, ***P<0.001, ablation versus control, *t*-test). (**d**,**e**) Measurement of mechanical allodynia in the two temporal models of ablation (**d**) DT3&5 group (*n*=12 for DT3&5 and 7 for control) and (**e**) DT7&9 (*n*=8 for DT7&9 and 8 for control). ***P<0.001, **P<0.01, ablation versus control ipsilateral side, *U*-test. Data represent mean±s.e.m. CX_3_CR1^CreER/+^:R26^iDTR/+^ mice with DT only were considered to be controls and mice with both TM+DT treatment was ablation group.

To directly assess the role of CX_3_CR1^+^ cells in the development and initiation of neuropathic pain, we administered DT (i.p., 50 μg kg^−1^) at POD3 and POD5 (DT3&5) after SNT and then examined the depletion of microglia and macrophages, as well as chronic pain behaviours (Fig. 3a). As expected, DT at POD3 and 5 completely ablated spinal microglia (Fig. 3b,c) and most DRG macrophages at POD7 (Supplementary Fig. 6). Interestingly, behavioural experiments revealed that mechanical allodynia was significantly reversed at POD7 (Fig. 3d). In addition, the reduced mechanical allodynia was sustained, lasting up to POD14 (Fig. 3d), although microglia had largely repopulated the spinal cord by this stage (data not shown). Consistently, thermal hyperalgesia was also significantly reversed when CX_3_CR1 cells were ablated at POD3 and POD5 after SNT (Supplementary Fig. 7a,b). These results indicate that CX_3_CR1^+^ cells are critical during the initiation of neuropathic pain.

Next, we asked whether CX_3_CR1^+^ cells are required for the maintenance of neuropathic hypersensitivity. To this end, we ablated microglia and macrophages when neuropathic pain was fully developed. DT was administered (i.p., 50 μg kg^−1^) at POD7 and POD9 (DT7&9), which completely depleted spinal microglia and most DRG macrophages at POD11 (Fig. 3b,c and Supplementary Fig. 6). Surprisingly, when we examined the pain-like behaviours in these mice, we found that mechanical allodynia was only transiently reversed but then maintained to a comparable level as those without CX_3_CR1^+^ cell ablation at POD11 (Fig. 3e). In addition, the mechanical allodynia persisted at least to POD14 in those mice with CX_3_CR1^+^ cell ablation (Fig. 3e). Consistently, thermal hyperalgesia in mice, in which CX_3_CR1^+^ cells were ablated at POD7 and 9, was also only transiently reversed compared with those in control mice without cell ablation (Supplementary Fig. 7a,c). Interestingly, in female mice, thermal hyperalgesia but not mechanical allodynia was transiently reversed after DT injection at POD7 and POD9 (Supplementary Fig. 8), suggesting that sexual dimorphism may differentiate CX_3_CR1^+^ cell's function in different modalities of neuropathic pain. Taken together, our results suggest that CX_3_CR1^+^ cells participate in the initiation of the neuropathic pain state, but are only transiently required in the maintenance of neuropathic pain. In particular, there during a critical time window of at POD0–POD5, when CX_3_CR1^+^ cells promote the transition from acute to chronic pain after peripheral nerve injury.

### Depletion of monocytes did not alter hypersensitivity

**Figure 4:**
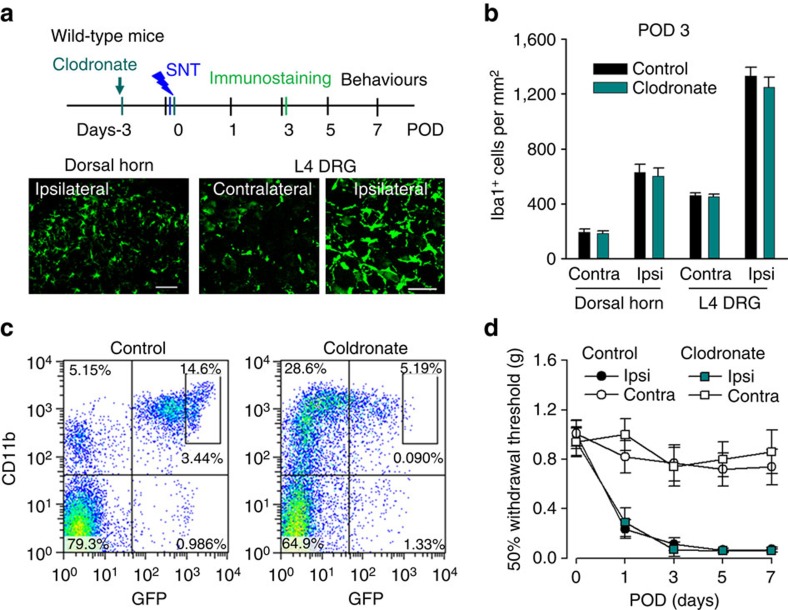
Depletion of peripheral monocytes does not affect neuropathic pain development. (**a**) An experimental diagram showing the timeline of drug treatments, SNT surgery, immunostaining and behavioural assays to test the role of peripheral monocytes in neuropathic pain. Representative images showing that the number of Iba1-positive spinal microglia and DRG macrophages in clodronate-treated group (C57BL/6 mice; lower). Scale bar, 50 μm. (**b**) Statistical data showing the density of dorsal horn microglia and DRG macrophages in control (C57BL/6, no treatment) and clodronate (C57BL/6, clodronate treatment) groups at POD3 SNT (*n*=3 mice for each group, *n*=3 images for each animal). (**c**) Representative blood cytometry data from CX_3_CR1^GFP/+^ mice showing CD11b^+^CX_3_CR1^+^ population at 3 days after clodronate treatment (*n*=3 for each group). (**d**) Behavioural measurements showing mechanical allodynia in clodronate (C57BL/6, clodronate treatment) and control (C57BL/6, no treatment) after SNT (data represent mean±s.e.m., *n*=8 for clodronate group and 7 for control group; *P*>0.05 for all testing points, *U*-test).

CX_3_CR1 is expressed in CNS microglia, DRG macrophages and circulating monocytes^26^. Our above results demonstrated the pivotal function of CX_3_CR1^+^ cells in neuropathic pain. However, the respective role of peripheral monocytes and microglia in neuropathic hypersensitivity remains unknown. To address this question, clodronate liposomes were used to deplete blood and tissue phagocytes including monocytes and macrophages^33^,^34^ (Fig. 4a). In wild-type mice, Iba-1 positive cells (presumably resident macrophages) in the spleen and liver were completely depleted after clodronate treatment (Supplementary Fig. 9). However, clodronate liposomes were unable to deplete spinal microglia (Fig. 4a,b). The DRG macrophages were also preserved after the treatment of clodronate liposomes (Fig. 4a,b), suggesting that either clodronate liposomes was not able to penetrate into the DRG, or macrophages in the DRG were unable to phagocytose clodronate liposomes. We tested the effect of clodronate liposomes (16 ml kg^−1^, i.p. twice at 3 days intervals) on blood CX_3_CR1^+^ cell depletion using CX_3_CR1^GFP/+^ mice. Indeed, CD11b^+^CX_3_CR1^+^ cells were largely ablated in the blood 2 days after the last clodronate liposome treatment (Fig. 4c). We further confirmed the depletion of blood monocytes by clodronate at POD3 after SNT using F4/80 (mature macrophage marker) and Gr-1 (also known as Ly-6, a marker of monocytes, neutrophils and eosinophils) staining in flow cytometry (Supplementary Fig. 10a,b). The results show that the Gr-1^low^ F4/80^high^ cells that correspond to mature blood monocytes^35^ were reliably depleted by clodronate liposomes. However, there was increased CD11b^+^CX_3_CR1^−^F4/80^low^Gr-1^high^ population after clodronate treatment, which might be repopulated monocytes^36^. These results show that application of clodronate liposomes depleted CD11b^+^CX_3_CR1^+^ monocytes but not DRG macrophages or spinal microglia.

It has been reported that blood monocytes can infiltrate the spinal dorsal horn and contribute to pain-like hypersensitivity after peripheral nerve injury^20^. Here we wanted to directly address the role of peripheral monocytes in neuropathic pain by testing pain-like behaviours after clodronate liposome treatment. Clodronate liposomes (16 ml kg^−1^, i.p.) were applied to wild-type mice at 3 days before and immediately after SNT. Interestingly, we found that clodronate liposome treatment did not alter the mechanical allodynia (Fig. 4d) or thermal hyperalgesia after SNT (Supplementary Fig. 10c). There was no difference between control mice and the clodronate liposome-treated mice in the development of hypersensitivity after peripheral nerve injury. These results indicate that circulating monocytes are not required for neuropathic pain induction. Therefore, the abolished pain-like hypersensitivity in CX_3_CR1^+^ cell-depleted mice might be due to the loss of CNS microglia and/or DRG resident macrophages.

### Ablation of resident microglia delays hypersensitivity

**Figure 5:**
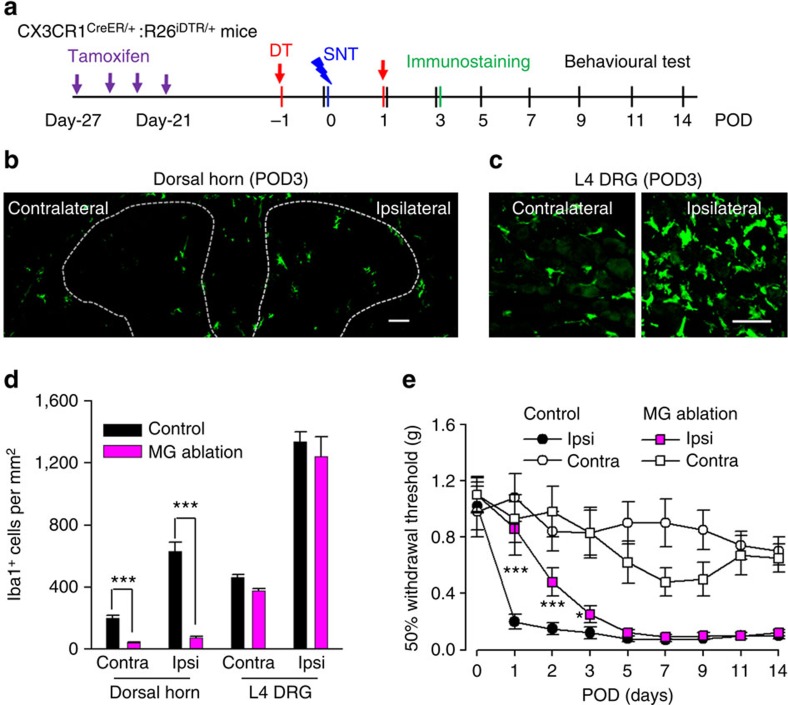
Microglia depletion delays but does not prevent neuropathic pain development. (**a**) An experimental diagram showing the timeline of drug treatments, SNT surgery and behavioural assays to test the role of resident microglia in SNT-induced neuropathic pain. TM was injected 3 weeks before DT treatment and SNT surgery. (**b**) A representative image showing microglia ablation in both contralateral and ipsilateral dorsal horn at POD3 after SNT. Scale bar, 100 μm. (**c**) Representative images showing macrophages in both contralateral and ipsilateral DRGs at POD3 after SNT. Scale bar, 50 μm. (**d**) Statistical data showing ablation effects at POD3 in the dorsal horn and DRG (*n*=3–4 mice for ablation and control group, *n*=2–3 images for each animal, ***P<0.001, *t*-test). MG, microglia. (**e**) Behavioural measurement showing mechanical allodynia in microglia ablation and control group (data represent mean±s.e.m., *n*=11 for ablation group and 8 for control group, ***P<0.001, *P<0.05, MG ablation ipsi versus control ipsi, *U*-test). CX_3_CR1^CreER/+^:R26^iDTR/+^ mice with DT only were considered to be controls and mice with both TM+DT treatment was MG ablation group.

Peripheral monocytes are reported to infiltrate the spinal cord to participate in hypersensitivity after peripheral nerve injury^20^. To further dissect the respective role of peripheral monocytes and resident microglia in neuropathic pain, we again used CX_3_CR1^CreER/+^:R26^iDTR/+^ mice, which enabled us to exclusively deplete resident microglia in the CNS but not blood CX_3_CR1^+^ cells. This is because resident microglia show a much slower turnover^37^, whereas blood CX_3_CR1^+^ cells have substantially rapid turnover and are replenished frequently^27^. To this end, we applied TM (i.p., 150 mg kg^−1^) to CX_3_CR1^CreER/+^:R26^iDTR/+^ mice and allowed a 3-week interval of peripheral turnover before DT injection (Fig. 5a). We found that spinal microglia at POD3 after SNT were largely depleted (Fig. 5b,d), with efficiencies comparable to the total CX_3_CR1^+^ cell depletion. However, macrophages in L4 DRG in these mice were similar to non-depleted control mice at POD3 (Fig. 5c,d).

Also, blood CD11b^+^ CX_3_CR1^+^ cells were largely preserved (Supplementary Fig. 11a,b). Therefore, it seemed that a 3-week interval between TM and DT application was able to selectively deplete CNS microglia but not blood CX_3_CR1^+^ monocytes and DRG macrophages in CX_3_CR1^CreER/+^:R26^iDTR/+^ mice. Since the depletion of monocytes by clodronate did not affect neuropathic pain development (Fig. 4), we surmised that microglial ablation might replicate the reversed pain phenotype in CX_3_CR1^+^ cell-depleted mice (Fig. 1). To test this idea, we examined the pain behaviours in these CX_3_CR1^CreER/+^:R26^iDTR/+^ mice with resident microglial ablation after SNT. To our surprise, we found that mice without microglia showed attenuated pain-like hypersensitivity only at POD1–3 (Fig. 5e). From POD5–14, the mechanical allodynia gradually returned to the similar level as those control mice with normal resident microglia (Fig. 5e). Consistently, thermal hyperalgesia in the microglial ablation mice after SNT were only reduced at POD1–3 but not at POD5–14 (Supplementary Fig. 11a,c). To test the possibility that this delayed hypersensitivity might be due to the potential secondary inflammation after microglia ablation, we examined the inflammatory cytokines interleukin (IL)-1β in control and microglial ablation mice using western blot. We found that IL-1β expression was not altered after microglia ablation in sham control. Also, IL-1β expression was equally upregulated at POD3 after SNT in mice with or without microglial ablation (Supplementary Figs 12a,b and 14). These results suggest that secondary inflammation after microglia ablation may not explain the delayed hypersensitivity in microglia-depleted mice. Interestingly, we found astrocyte marker glial fibrillary acidic protein (GFAP) was significantly increased after microglial ablation in sham control and also at POD3 after SNT (Supplementary Figs 12a,c and 14). Nevertheless, our results unexpectedly found that the selective depletion of CNS microglia delayed but did not reverse neuropathic hypersensitivity after peripheral nerve injury.

### Monocytes do not infiltrate into the dorsal horn after SNT

**Figure 6:**
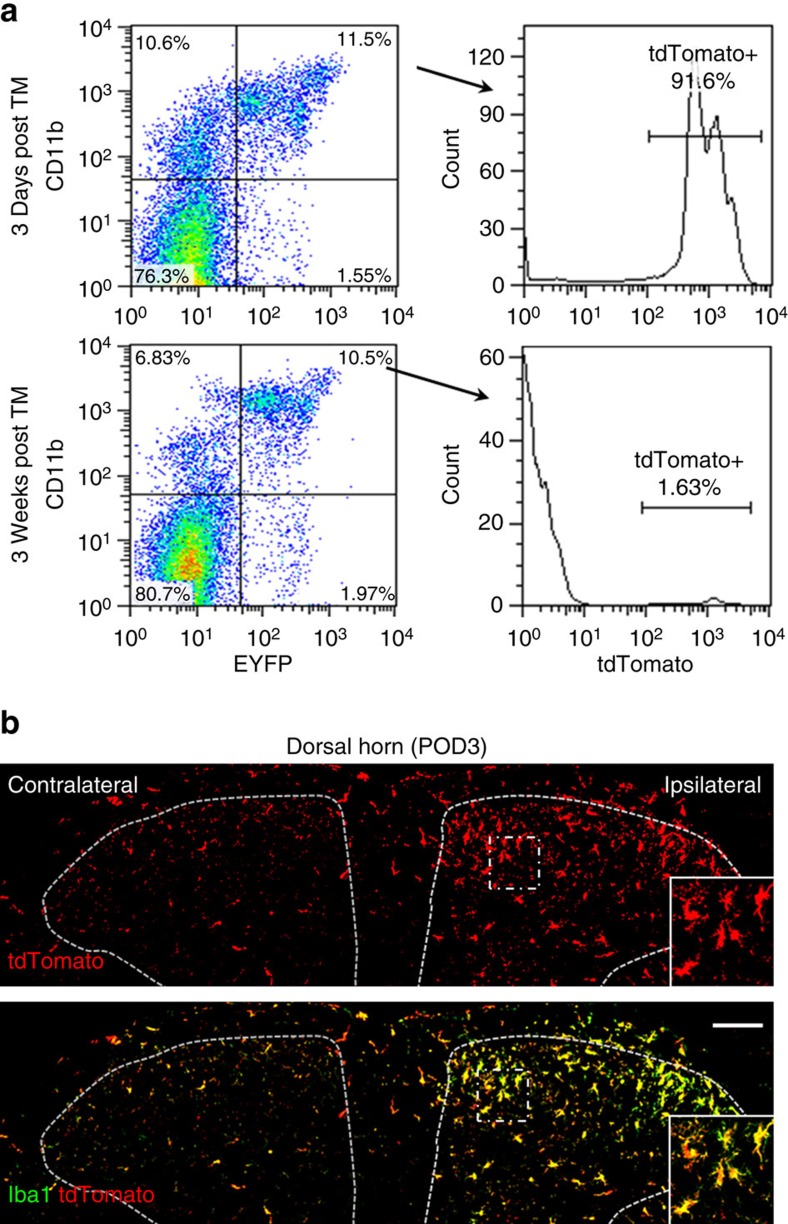
There is no infiltration of blood monocytes in the dorsal horn after SNT. (**a**) Representative blood cytometry data from CX_3_CR1^CreER/+^/R26^tdTomato/+^ reporter mice showing the CD11b^+^/CX_3_CR1^+^ population at 3 days and 3 weeks post tamoxifen administration (*n*=3 for each time point). (**b**) Iba1 staining (green) showing all spinal dorsal horn microglia were tdTomato (red) positive at POD3 after SNT in CX_3_CR1^CreER/+^/R26^tdTomato/+^ mice with 3-week interval between TM and DT treatment. The results indicate no peripheral monocyte infiltration to spinal cord at POD3 after SNT. Higher-magnification images from boxed regions in larger images showing microglial morphology (*n*=3 mice for each group, *n*=3 images for each animal). Scale bar, 100 μm.

The recovery of pain-like hypersensitivity after SNT in CNS microglia-ablated mice could be due to the monocyte infiltration since peripheral monocytes are known to infiltrate into spinal cord after bone marrow transplantation^20^. However, the 3-week interval between TM and DT application in CX_3_CR1^CreER/+^:R26^iDTR/+^ mice was able to largely deplete the spinal microglia after SNT (Fig. 5b,d), suggesting that there was no infiltration of peripheral monocytes into the spinal cord after peripheral nerve injury. To further examine the possibility of monocyte infiltration after SNT, we crossed a cre-inducible RFP (tdTomato) reporter (R26^RFP/+^) mice with CX_3_CR1^CreER/+^ mice to generate CX_3_CR1^CreER/+^:R26^RFP/+^ mice. We then monitored tdTomato^+^ cells that are CX_3_CR1^+^ either at 3 days or 3 weeks after TM treatment. As expected, we found that <2% of CD11b^+^ CX_3_CR1^+^ monocytes were labelled with tdTomato 3 weeks after TM treatment, while over 90% of CD11b^+^ CX_3_CR1^+^-positive cells were tdTomato positive at 3 days after TM treatment (Fig. 6a). Therefore, blood CX_3_CR1^+^ cells were indeed replaced within a 3-week period.

We then examined the monocyte infiltration in CX_3_CR1^CreER/+^:R26^RFP/+^ mice after SNT. If there was monocyte infiltration, we would have observed Iba1^+^ tdTomato^−^ cells in the ipsilateral spinal cord after SNT. However, we found that at POD3 after SNT, all microglia stained with Iba1 in both ipsilateral and contralateral dorsal horn were also labelled with tdTomato (Fig. 6b). These results further confirm that there is no infiltration of blood monocytes to the spinal dorsal horn after SNT in our study.

### Synergistic action of microglia and monocytes

**Figure 7:**
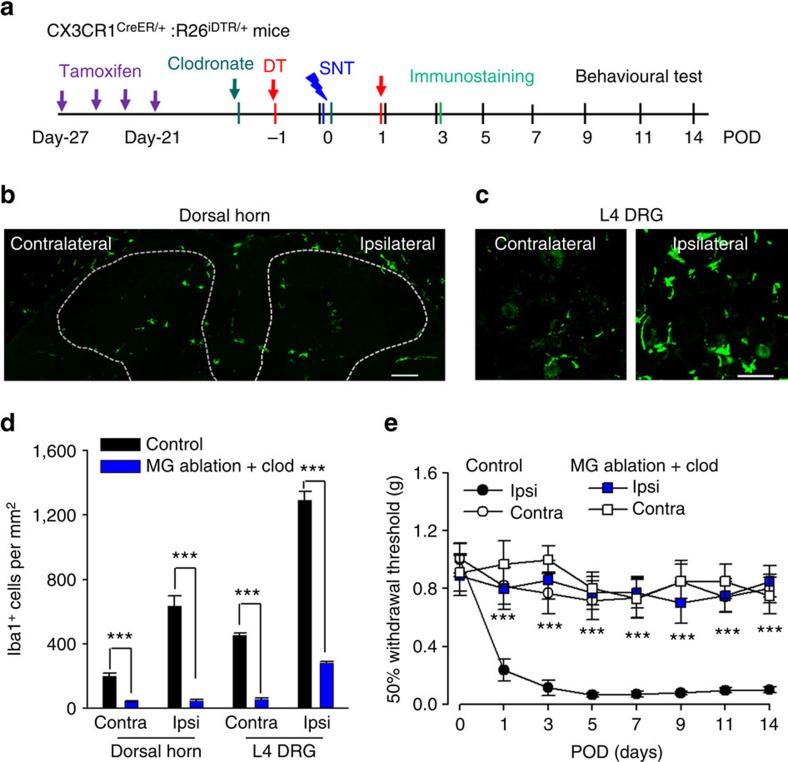
Microglia ablation plus peripheral monocyte depletion prevent neuropathic pain development. (**a**) An experimental diagram showing the timeline of drug treatments, SNT surgery and behavioural tests. Clodronate was injected before DT injection. (**b**) Confocal images showing Iba1 staining in the spinal dorsal horn at POD3 in control and ablation mice. Scale bar, 100 μm. (**c**) Confocal images showing depletion of L4 DRG macrophages at POD3. Scale bar, 50 μm. (**d**) Statistical data showing the number Iba1+ cells in the dorsal horn and DRG in control and ablation group (*n*=3 mice for each group, *n*=2–3 images for each animal, ***P<0.001, *t*-test). (**e**) Behavioural assessment showing mechanical allodynia in mice with microglia ablation plus monocyte depletion (data represent mean±s.e.m., *n*=8 for ablation and 7 for control group. ***P<0.001, *U*-test). CX_3_CR1^CreER/+^:R26^iDTR/+^ mice with DT only were considered to be controls and mice with TM+DT+clodronate treatment was ablation group.

So far, we have shown that depletion of CX_3_CR1^+^ cells abolished neuropathic pain (Fig. 1), while peripheral monocytes or microglia depletion did not affect (Fig. 4) or only delayed the development of pain-like hypersensitivity after SNT (Fig. 5), respectively. These results strongly suggest that both resident microglia and monocytes are required for the full development of neuropathic pain. To test this idea, we applied clodronate liposomes to deplete monocytes in mice with ablated resident microglia, and tested their pain behaviours. Clodronate liposomes were applied 3 days before and immediately after SNT while DT was administered at 1 day before and POD1 after SNT in CX_3_CR1^CreER/+^:R26^iDTR/+^ mice (Fig. 7a). With clodronate and DT treatment, spinal microglia (Fig. 7b,d), DRG macrophages (Fig. 7c,d) and blood CX_3_CR1^+^ cells (data not shown) were mostly depleted at POD3 after SNT. Our data show that DRG macrophage depletion did not occur in both our peripheral (Fig. 4c) and central (Fig. 5c) ablation models. However, the number of DRG macrophages was drastically reduced in our combined ablation model (Fig. 7c). These results suggest that DRG macrophages are CX_3_CR1^+^ and are slowly replenished by blood monocytes (data not shown). Therefore, clodronate depleted the infiltration source (blood monocytes), resulting in an overall reduction in number of DRG macrophages. Future investigations on the specific contribution of DRG macrophages to neuropathic pain are warranted.

Next, we examined pain behaviours in CX_3_CR1^CreER/+^:R26^iDTR/+^ mice, with both clodronate and DT treatment. Consistent with those in the total CX_3_CR1^+^ cell-depleted mice (Fig. 1e), we found that both mechanical allodynia (Fig. 7e) and thermal hyperalgesia (Supplementary Fig. 13) after SNT were completely prevented after clodronate and DT treatment compared with control mice without the treatment. These results corroborate the data obtained from total CX_3_CR1^+^ cell-depleted mice, indicating that both peripheral monocytes and resident microglia are required for the development of neuropathic pain. Therefore, the two populations of CX_3_CR1^+^ cells may synergistically promote the transition from acute pain to chronic pain after peripheral nerve injury.

## Discussion

Currently available treatments for chronic neuropathic pain typically show limited efficacy in a majority of patients^38^. Therefore, comprehensive studies of chronic pain pathogenesis are required to identify novel therapeutic targets and develop a better treatment for neuropathic pain. It is well known that spinal microglia and peripheral monocytes contribute to neuropathic pain development after peripheral nerve injury. Indeed, systemic inhibition of microglia and macrophages by the broad inhibitor minocycline, attenuated neuropathic pain behaviours^39^,^40^. However, the temporal and spatial function of resident microglia and peripheral monocytes in the development of neuropathic pain has not been elucidated. Using CX_3_CR1^CreER/+^:R26^iDTR/+^ mice, combined with clodronate treatment enabled us to deplete microglia and monocytes in a controllable fashion. We discovered a critical time window for microglia and monocytes in promoting the transition from acute to chronic pain after peripheral nerve injury. Moreover, we demonstrated that resident microglia and peripheral monocytes synergistically initiate pain-like hypersensitivity after peripheral nerve injury. These novel findings provide a rationale for targeting both microglia and macrophages to prevent the development of neuropathic pain. Our understanding of the exact role of microglia and monocytes in the initiation and maintenance of neuropathic pain was limited due to our inability to manipulate these cells in a temporally distinct manner. To circumvent this limitation, we took advantage of CX_3_CR1^CreER/+^:R26^iDTR/+^ mice that expresses inducible DTR selectively in CX_3_CR1^+^ cells, including CNS resident microglia, peripheral macrophages and a subset of circulating monocytes. Thus, we are able to ablate CX_3_CR1^+^ cells at different time points during the development and maintenance of neuropathic pain. We found that pain-like hypersensitivity after SNT was reversed when CX_3_CR1^+^ cells were depleted at early (POD0–5) but not at late time points (POD7–9) after SNT. These results provide direct evidence showing that CX_3_CR1^+^ cells participate in the initiation, however, during the maintenance phase, microglia-independent pathways may take over in the absence of microglia. Consistent with this notion, previous studies found that minocycline, a broad microglia and macrophage inhibitor, attenuated the development but not the existing hypersensitivity after peripheral nerve injury^39^,^40^. In addition to inhibiting proinflammatory enzymes, minocycline directly targets DRG sodium channels, matrix metalloproteinases (MMPs) and scavenges reactive oxygen species (ROS)^41^,^42^,^43^. Particularly, the nonspecific action of minocycline on neurons other than microglia makes it difficult to draw meaningful conclusions using the drug^43^,^44^,^45^,^46^. Our current study directly addressed the question by depleting CX_3_CR1^+^ cells and pinpointed a critical time window for microglia and monocytes in the development of neuropathic pain.

Intriguingly, our data show that the reduction in pain-like hypersensitivity in our ablation regimes was long-lasting even after microglia repopulation. These results suggest a critical period during which microglia are essential for the induction of neuropathic pain and during which the absence of microglia prevents the overall development of pain. There are at least two possible explanations why pain-like hypersensitivity was not restored in the presence of repopulated microglia: (1) critical signals that generated during peripheral nerve injury were missing when microglia were repopulated; and (2) the repopulated microglia were not able to respond to pain stimulation. However, since our results show that repopulated microglia have the capability to engage in the neuropathic pain induction (Fig. 2), we propose that critical signals for microglial activation, such as CSF-1 or ATP^30^,^32^, during the induction of neuropathic pain were not present or functional at a later time when microglia were repopulated. Consistent with this notion, we found that mice treated with CSF-1-neutralizing antibody failed to develop neuropathic hypersensitivity that was also long-lasting even after the administration of CSF-1 antibody was terminated (Supplementary Fig. 4). Therefore, our study indicates a critical time window during which microglia/monocytes are essential for the induction of neuropathic pain and absence of microglia/monocytes during this critical window prevents neuropathic pain development.

Our results also show that although microglia and monocytes initiate pain-like hypersensitivity they are not that critical for the maintenance of pain-like hypersensitivity after peripheral never injury. Consistently, there is a rapid spinal microglial response in various rodent models of neuropathic pain, including morphological change, proliferation, functional upregulation of molecules such as Nox2, P2Y12, P2X4 and CSF1R, and release of mediators such as tumour necrosis factor-α, IL-1β and brain-derived neurotrophic factor^30^,^47^,^48^,^49^. These microglial responses to peripheral nerve injury dwindle after a week although they still remain significantly upregulated compared with the control condition without nerve injury. In line with the temporal activation profile of microglia, it has been reported that MAPK ERK phosphorylation is sequentially increased in spinal neurons, microglia and astrocytes. Particularly, p-ERK expression is widespread in microglia with peaks between POD1 and POD3, but by POD21 its expression is predominantly localized to astrocytes in the dorsal horn despite the persistently high density of microglia^50^. Taken together, it seems that MAPK signalling and its downstream effector(s) may underlie the temporal role of microglia in the initiation, but are not as critical for maintenance of neuropathic pain. Interestingly, a recent study found that sulforaphane, a ROS scavenger, attenuated neuropathic pain during the early phase but was ineffective at the late phase when allodynia was established^51^. This is in line with our study, since sulforaphane mainly inhibits spinal microglia-derived ROS production and macrophage NOX2 in DRGs after nerve injury^51^,^52^.

A recent report showed that sexual dimorphism plays an important role in mechanical hypersensitivity^28^. Interestingly, we found gender differences in mechanical allodynia (but not in thermal hyperalgesia) during the pain maintenance phase but not during the initiation phase (Supplementary Figs 3 and 8). These results suggest that microglia equally participated in the mechanical pain development in both male and female mice. Moreover, our results suggest that sexual dimorphism may play a differential role in the different pain modalities. The significance and underlying mechanism for the role of gender differences in pain initiation versus maintenance warrants further investigation.

Compared with the well-documented function of microglia in neuropathic pain, the role of monocytes in neuropathic pain is still uncertain. Clodronate depletion of macrophages and monocytes reduced thermal hyperalgesia and Wallerian degeneration in one study^22^ although the same treatment had no effect on mechanical allodynia in another study^21^. In contrast, a recent study using bone marrow transplantation provided support to the idea that circulating blood monocytes are able to infiltrate into the spinal cord and thus contribute to neuropathic pain via central sensitization^20^. In our study, we found that sole depletion of peripheral macrophages had a limited effect on pain-like hypersensitivity. With liposomal clodronate treatment, we could specifically deplete most CX_3_CR1^+^ monocytes from the blood but not CNS microglia. Under this condition, the mice without blood monocytes developed neuropathic pain normally after SNT. Moreover, we have strong evidence showing that there was no infiltration of peripheral microglia in the spinal cord after peripheral nerve injury. Therefore, the infiltrated monocytes observed in the study could be due to the irradiation chimerism protocol that may lead to non-physiological transmigration of cells into the spinal cord^37^. However, we cannot exclude the possibility that the CX_3_CR1^CreER/+^:R26^RFP/+^ mice used in this study lack a copy of the endogenous CX3CR1 gene and may account for the lack of monocyte infiltration observed. Our study suggests that even without peripheral monocytes, resident microglia are sufficient to initiate neuropathic pain. However, we were surprised that depletion of resident microglia did not abolish, but only delayed the development of neuropathic pain. Hence, peripheral monocytes are capable of playing an equally important role in initiating neuropathic pain and are able to help facilitate neuropathic pain even in the absence of resident microglia. Together, our results strongly indicate that microglia and monocytes synergistically promote the transition from acute to chronic pain after peripheral nerve injury. Indeed, we found that neuropathic pain-like hypersensitivity was completely reversed in CX_3_CR1^+^ cell-ablated mice and in the microglia-ablated mice treated with clodronate liposomes. Future studies are needed to address the molecular mechanisms underlying synergistic interaction between resident microglia and peripheral monocytes in gating the neuropathic hypersensitivity.

Our results are novel in that we were able to dissect the respective roles of resident microglia and peripheral monocytes in neuropathic pain using a combination of genetic and pharmacological tools. The limited effect of microglial ablation on pain behaviours was unexpected, considering that numerous studies have proved the critical function of microglia in neuropathic pain. However, since most studies were not able to distinguish the particular molecules in microglia versus monocytes and macrophages, the interpretation of microglial function in those studies should be interpreted with caution. Although our monocyte depletion study indeed supports the important function of microglia, we believe that both microglia and monocytes work in concert to initiate neuropathic pain after nerve injury. However, peripheral monocytes alone may be capable of initiating pain under conditions where microglial activation is minimal^23^,^25^. A caveat is that we also found astrocyte activation in the spinal cord after microglial depletion. This result may complicate the explanation for the delayed pain-like hypersensitivity caused by monocytes alone, considering the critical role of astrocytes in neuropathic pain maintenance^5^,^53^. However, since there is no obvious pain-like hypersensitivity despite the astrocyte activation in microglia ablation mice in sham control, we suspect that the reactive astrocytes may not directly account for delayed hypersensitivity after microglia depletion. In sum, our current study demonstrates that microglia and monocytes participate in the initiation of pain-like hypersensitivity but may not be as crucial for its maintenance after peripheral nerve injury. In addition, either resident microglia or peripheral monocytes are sufficient to initiate neuropathic pain and thus they synergistically promote the transition from acute to chronic neuropathic pain. Our results provide a rationale for early intervention of pain development targeting both resident microglia and peripheral monocytes.

## Methods

### Animals

Mice (7–12 weeks old) were used in accordance with institutional guidelines as approved by the animal care and use committee at Rutgers University. C57BL/6J (Charles River) and CX_3_CR1^GFP/+^ mice were used as wild-type control. CX_3_CR1^CreER−EYFP/+^ mice were obtained from Dr Wen-Biao Gan at New York University. The mice were crossed with R26^iDTR/+^ or R26^tdTomato/+^ (purchased from Jax lab) to obtain CX_3_CR1^CreER/+^:R26^iDTR/+^or CX_3_CR1^CreER/+^:R26^tdTomato/+^ mice, respectively. Male mice were used throughout the study, unless, the use of female mice was specifically indicated. Mice were assigned to experimental groups randomly within a litter. Experimenters were blind to drug treatments.

### Surgery

Lumbar 4 SNT was done in 7- to 9-week-old mice. SNT surgery was performed under 2% isoflurane anaesthesia. An incision was made along the mid-line of the lumbar spine. The left paraspinal muscles in front of the pelvic bone were separated to expose the L5 transverse process. The L5 transverse process was removed to expose L4 spinal nerve. The L4 spinal nerve was separated and transected and removed 1–1.5 mm from the end to DRG. The wound was then irrigated with sterile PBS and closed with #6 silk sutures for the muscles and #5 silk sutures for the skin. POD represents the post-operative day following SNT and all the experimental timelines are in reference to POD0, which is the day of SNT surgery.

### CX_3_CR1^+^ cell ablation

TM (Sigma) was administered as a solution in corn oil (Sigma) to mice over 6 weeks old by i.p. injection. Animals received four doses of TM (150 mg kg^−1^, 20 mg ml^−1^ in corn oil) in 48-h intervals. For total CX_3_CR1^+^ cell ablation, two doses of DT (Sigma, Catalogue #D0564, 50 μg kg^−1^, 2.5 μg ml^−1^ in PBS) were given at 3 and 5 days after the last TM treatment. For microglia ablation, the interval between the last TM and the first DT was 3 weeks. Mice administered with DT only (without TM) were used as control for all ablation experiments.

### Monocyte depletion

Liposome-encapsulated clodronate was used to deplete phagocytic macrophages. Clodronate liposomes (15 ml kg^−1^, ClodronateLiposomes.com) were i.p. injected 3 days before and immediately after the SNT surgery.

### CSF-1-neutralizing antibody treatment

CSF-1 antibody (200 ng in 5 μl ACSF, R&D #AF416) or the vehicle (ACSF) were daily injected intrathecally by direct lumbar puncture between L5 and L6 vertebrae of the spine, using a 10-μl Hamilton syringe (Hamilton Bonaduz AG) with a 31G needle. Successful lumbar puncture was identified by tail reflex.

### Behavioural measurement

Mechanical allodynia was assessed by measuring the paw withdraw threshold, with a set of Von Frey filaments (0.04–2 g; North Coast medical). Mice were placed on an elevated metal grid. The filament was applied to the plantar surface at a vertical angle for up to 3 s from the bottom. Fifty per cent withdraw threshold values were determined using the up–down method^54^.

Thermal hyperalgesia was assessed by measuring the paw withdraw latency to radiant heat stimuli. Mice were placed in elevated chambers with Plexiglas floor and allowed to habituate for 20 min. The radiant heat source (IITC Inc life science) was applied to the centre of the plantar surface of the hind paw four times with at least 3-min intervals. The average withdrawal latency of the four trials was recorded as the response latency.

Tail flick was assessed using the same radiant heat source (IITC Inc life science). Mice were restrained in a cylindrical holder with the tail hung out. Heat was applied to the tail at ∼2 cm from the rear end. The machine automatically detects the flick latency as the light/heat beam travels unhindered after the tail moves away.

The rotarod tests were performed using a four-lane Rotarod apparatus (Med Assocaites Inc). The rotarod speed started from 4 rounds per minute and uniformly accelerated to 40 rounds per minute in 5 min. Each mouse was tested for 3 times with 5-min interval.

### Fluorescent immunostaining

Mice were deeply anaesthetized with isoflurane (5% in O_2_) and perfused transcardially with 20 ml PBS followed by 20 ml of cold 4% paraformaldehyde (PFA) in PBS containing 1.5% picric acid. The spinal cord and DRG were removed and post-fixed with the same 4% PFA for 4–6 h at 4 °C. The samples were then transferred to 30% sucrose in PBS overnight. Sample sections (14 μm in thickness) were prepared on gelatin-coated glass slide with a cryostat (Leica). The sections were blocked with 5% goat serum and 0.3% Triton X-100 (Sigma) in TBS buffer for 60 min, and then incubated overnight at 4 °C with primary antibody for rabbit-anti-Iba1 (1:1,000, Wako Chemicals, Catalogue #019-19741), rat-anti-CD11b (1:200, Biolegend, Catalogue #101202) and rabbit-anti-Ki-67 (1:500, Abcam, Catalogue #16667). The sections were then incubated for 60 min at room temperature, with secondary antibodies (1:500, Alexa Fluor 594, Life Technologies). The sections were mounted with Fluoromount-G (SouthernBiotech) and fluorescent images were obtained with a confocal microscope (LSM510, Zeiss). Cell counting and fluorescent signal intensity was quantified using ImageJ software (National Institutes of Health, Bethesda, MD). Note that the EYFP signal in CX_3_CR1 cells in tissue obtained from CX_3_CR1^CreER/+^: R26^iDTR/+^ mice was too weak. Hence, Iba-1 staining was performed as described above. The Iba-1 staining images were represented in green channel (using Image J) for consistency.

### Monocyte flow cytometry

Whole mouse blood was collected and monocytes were separated from erythrocyte and granulocyte on a Ficoll (GE Healthcare) gradient. Separated monocytes were washed with Hank's Balanced Salt Solution and then incubated with 2% goat serum for 10 min, and single stained with allophycocyanin (APC)-conjugated CD11b antibody (1:200, Biolegend, Catalogue #101212) or double stained with CD11b plus PE-conjugated Gr-1antibody (1:600, Biolegend, Catalogue #108408) or PE-conjugated F4/80 antibody (1:100, Biolegend, Catalogue #123110) for 45 min. Cells were then fixed with 1% PFA for 10 min before flow cytometry. Cells population data were obtained on a FACS Calibur cytometer (Becton Dickinson) using the CellQuest software (Becton Dickinson). Data were analysed using FlowJo software (FlowJo, LLC). CX_3_CR1^CreER/+^:R26^iDTR/+^or CX_3_CR1^CreER/+^:R26^tdTomato/+^ mice were used for flow cytometry experiments. Both the EYFP and tdTomato signals were well detectable in the blood monocytes, hence no additional signal amplification strategy was used to detect these signals.

### Western blot

Under isoflurane anaesthesia, lumbar 4–5 spinal dorsal horn in ipsilateral were collected in different treatment groups of mice. The tissues were homogenized and sonicated on ice in SDS lysis buffer with protease inhibitor cocktail (Roche Molecular Biochemicals) and phosphatase inhibitor, followed by centrifugation at 13,000 r.p.m. for 20 min at 4 °C to obtain supernatant containing protein. Equal concentration of protein from different supernatant were loaded and separated by SDS–PAGE. After the transfer to a PVDF membrane (Bio-Rad), blots were blocked and incubated at 4 °C overnight with primary antibodies, rabbit anti-IL-1β (Abcam, 1:2,000), mouse anti-GFAP (Cell Signalling Technology, 1:2,000) and mouse anti-β-actin (Cell Signalling Technology, 1:2,000). Following which, the blots were incubated with horseradish peroxidase-conjugated goat anti-rabbit or goat anti-mouse IgG (secondary antibody, 1:5,000, Jackson Immune Laboratory) for 1 h at 25 °C and washed. The immune complex on the membrane was detected by SuperSignal West Femto Maximum Sensitivity Substrate (34,095; Thermo Scientific) and captured on ImageQuantLAS4000 (Fujifilm Life Science). Integrated optical density was determined using ImageJ 1.48 (NIH). Standard curves were constructed to establish that we operated within the linear range of the detection method.

### Statistical analysis

Quantification of Iba1 cells was done with ImageJ software (NIH Image). Data were presented as mean±s.e.m. Student's *t*-test and Wilcoxon rank-sum test (*U*-test) were used to establish significance. No statistical methods were used to predetermine sample sizes.

### Data availability

All relevant data and analysis are available on request from the corresponding author.

## Additional information

**How to cite this article:** Peng, J. *et al.* Microglia and monocytes synergistically promote the transition from acute to chronic pain after nerve injury. *Nat. Commun.* 7:12029 doi: 10.1038/ncomms12029 (2016).

## Supplementary Material

Supplementary Information Supplementary Figures 1-14
