# Supplementary material for: Microglia and monocytes synergistically promote the transition from acute to chronic pain after nerve injury
Source: Nat Commun. 2016 Jun 28;7:12029. doi: 10.1038/ncomms12029 (PMC4931235; doi:10.1038/ncomms12029)
Supplement: Supplementary Information — Supplementary Figures 1-14 [file ncomms12029-s1.pdf]

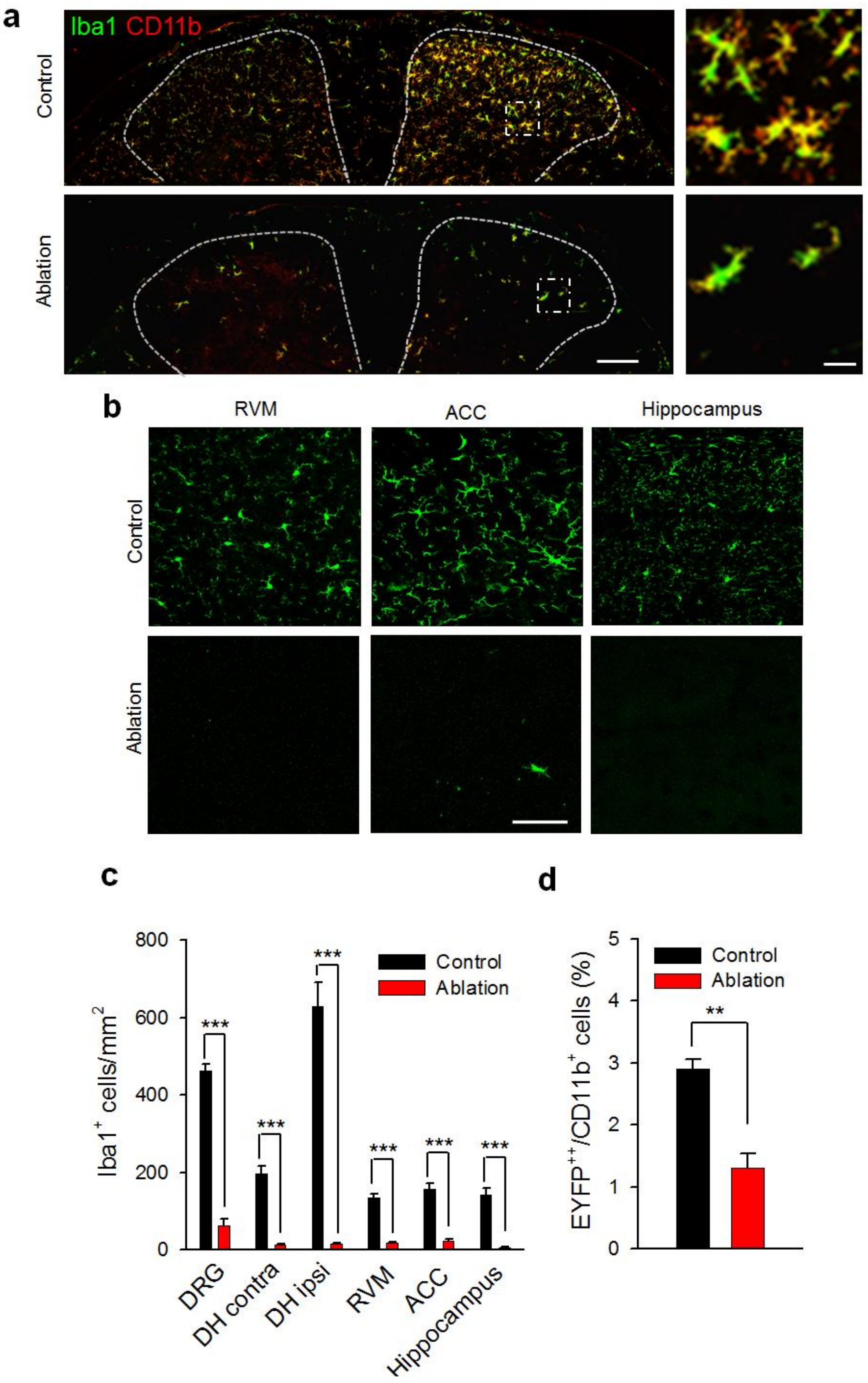

Supplementary Figure 1. CX_3CR1^+ cell ablation depletes brain microglia and a subset of blood monocytes. (a) Iba1 and CD11b double staining showing co-localization of iba1 and CD11b signals in all cells from the control mice and the

remained cells from the ablation mice at POD3. Scale bar: 100 µm. Right: Representative higher magnification images from boxed regions showing microglial morphology. Scale bar: 20 µm. **(b)** Iba1 staining showing microglia ablation in brain regions at 2 d after last DT injection. Scale bar, 50µm. **(c)** Statistical data showing the ablation efficiency in the DRG, dorsal horn (DH), rostral ventromedial medulla (RVM), anterior cingulated cortex (ACC) and hippocampus. **(d)** Statistical analysis showing that total CX_3CR1^+ cell ablation greatly reduced the percentage of $CD11b^+CX_3CR1^{high}$ monocytes population (n = 3 for each group, $**p < 0.01$, $***p < 0.001$, t-test). $CX_3CR1^{CreER/+}$:$R26^{iDTR/+}$ mice with DT only were considered to be controls and mice with both TM + DT treatment was ablation group.

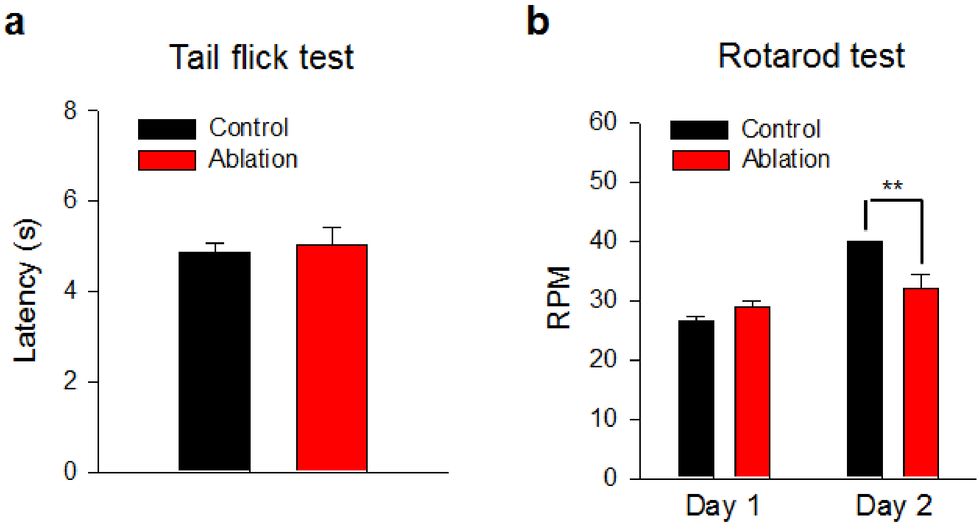

Supplementary Figure 2. CX_3CR1^+ cell ablation does not affect acute pain responses and basal motor function. (a) The tail flick response to a radio light stimulus in the CX_3CR1^+ cell ablation mice at 2 d after last DT injection was similar compared to the control mice. **(b)** Analysis of rotarod test showing that CX_3CR1^+ cell ablation did not affect the basal motor function but impaired the inter-session motor learning improvement. The same animals were used for both tail flick test and rotarod test (n = 6 for each group, ***p* < 0.01, t-test). $CX_3CR1^{CreER/+}$:$R26^{iDTR/+}$ mice with DT only were considered to be controls and mice with both TM + DT treatment was ablation group.

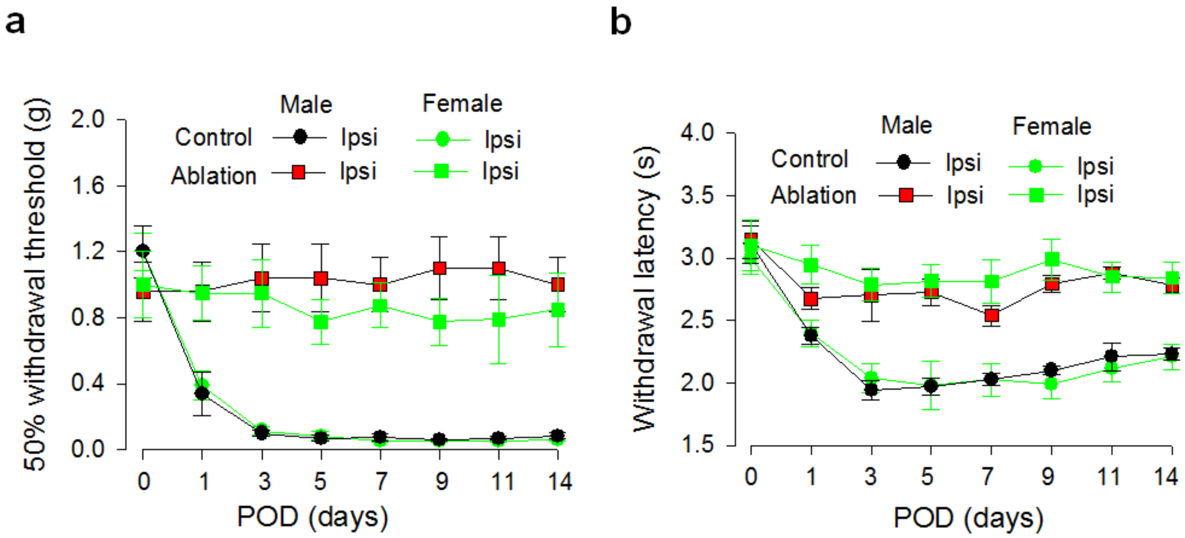

Supplementary Figure 3. CX_3CR1^+ cell ablation prevents neuropathic pain development in both male and female mice. The female mice of ablation group received the same DT treatment before and after SNT surgery as the male mice did. Behavioral assessment showing that both mechanical allodynia (**a**) and thermal hyperalgesia (**b**) were fully abolished in DT treated female mice similar to that seen in the male mice (n = 9 for male ablation group, n = 8 for female ablation group and n = 7 for each control groups, $p > 0.05$, for all testing points, male ablation vs. female ablation, U-test for mechanical and t-test for thermal responses, respectively). $CX_3CR1^{CreER/+}$:$R26^{iDTR/+}$ male and female mice with DT only were considered to be controls and mice with both TM + DT treatment was ablation group.

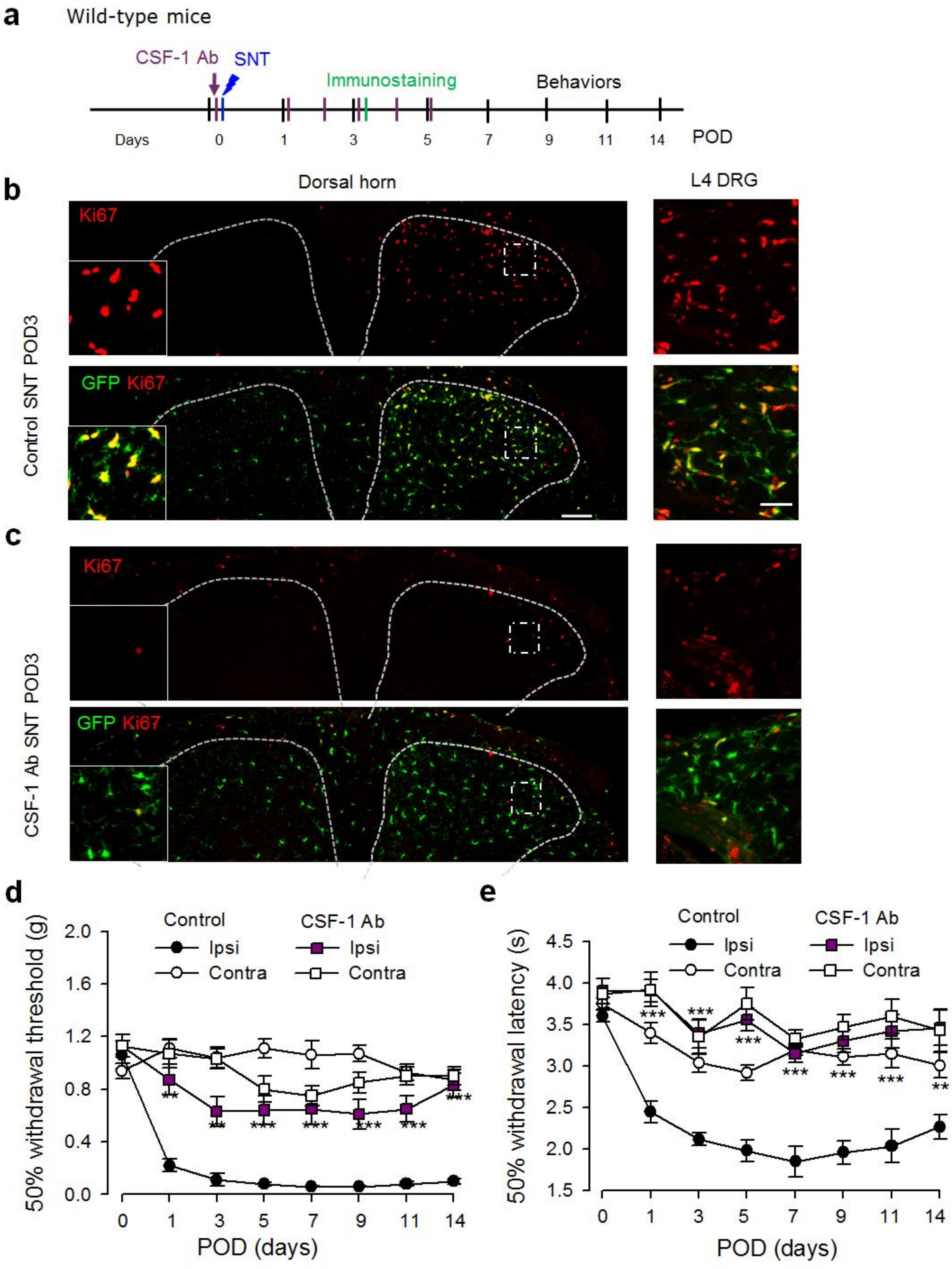

Supplementary Figure 4. CSF-1 signaling is required for microglia proliferation and pain development. (a) An experimental diagram showing the

timeline of drug treatments, SNT surgery and behavioral tests. CSF-neutralizing antibody was administered immediately before the SNT and at POD1-5. (**b-c**) Representative confocal images showing co-localization proliferation marker, Ki67, is shown in red in the spinal dorsal horn (Left) and ipsi DRG (right) in (**b**) control (vehicle- artificial cerebrospinal fluid) and (**c**) CSF-1 antibody treated $CX_3CR1^{GFP/+}$ mice, in which CX_3CR1^+ microglia are shown in green, at POD3 after SNT. Scale bar: Left, 100 µm, Right, 50 µm. (**d-e**) Pain behavior assessment showing (**d**) mechanical allodynia and (**e**) thermal hyperalgesia in control (vehicle) and CSF-1 antibody treated mice. (n = 9 for CSF-1 Ab, n = 7 for control group). $^{**}p < 0.01$, $^{***}p < 0.001$, U-test for mechanical allodynia and t-test for thermal hyperalgesia, respectively. These experiments were performed on wild-type mice.

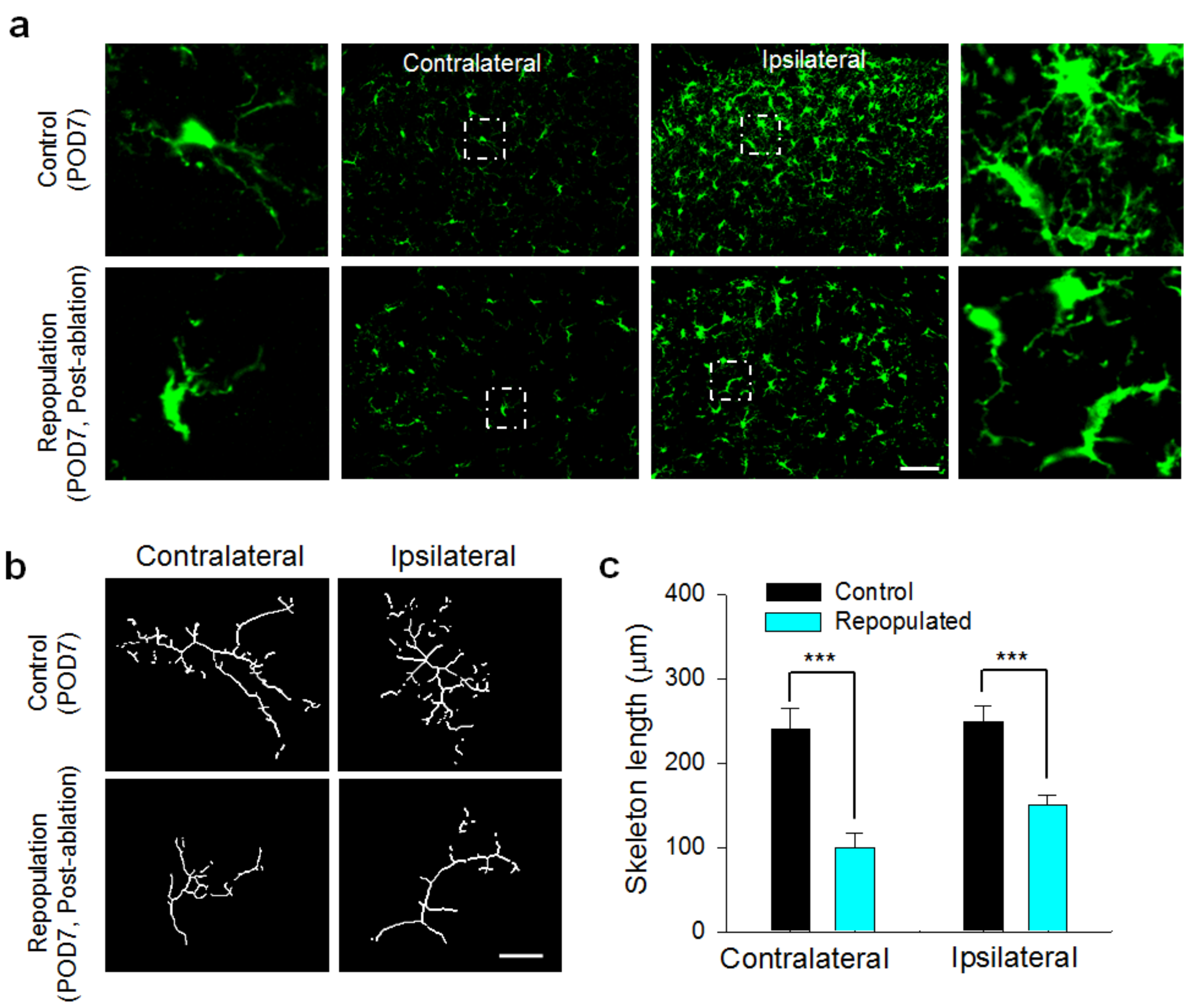

Supplementary Figure 5. Morphology of repopulated spinal microglia at POD7. (a) Iba1 staining showing that the spinal microglia were repopulated in both contralateral and ipsilateral dorsal horn. Scale bar: 50µm. Left and Right, high magnification of boxed area showing repopulated spinal microglia have less ramified and shorter processes compared to control microglia at POD7 after SNT. **(b)** Representative samples showing the skeletonized morphology of control and repopulated microglia. Scale bar: 10µm.**(c)** Statistical analysis showing that the skeleton lengths of repopulated microglia in both contralateral and ipsilateral dorsal horn were significantly shorter than the control microglia (n = 3 for each group, ***p < 0.001, t-test). Data were obtained from $CX_3CR1^{CreER/+}$:$R26^{iDTR/+}$ mice with DT (control) and TM + DT (ablation/ repopulation group).

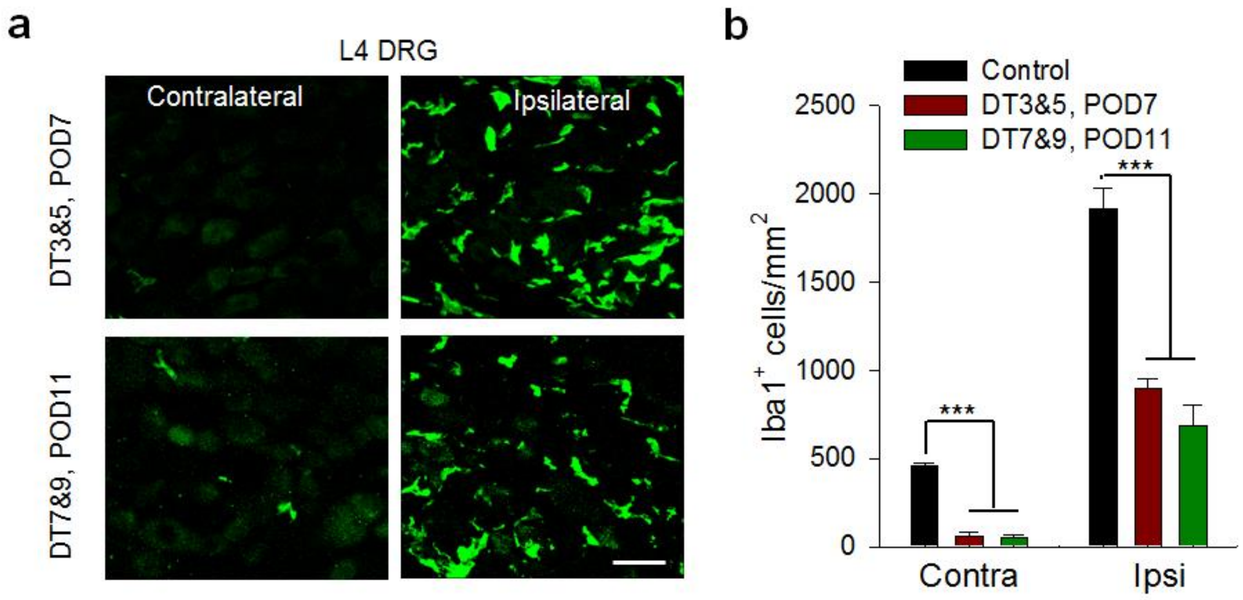

Supplementary Figure 6. Depletion of DRG macrophages with DT treatment. (a) Representative macrophage images using Iba1 staining in both contralateral and ipsilateral DRGs in $CX_3CR1^{CreER/+}$:$R26^{iDTR/+}$ mice treated with DT at POD3 and 5 after SNT (DT3&5) or at POD7 and 9 after SNT (DT7&9). Scale bar, 50μm. **(b)** Statistical analysis showing macrophage ablation in both contralateral and ipsilateral DRGs (n = 3-4 for each group, ***p < 0.001, t-test).

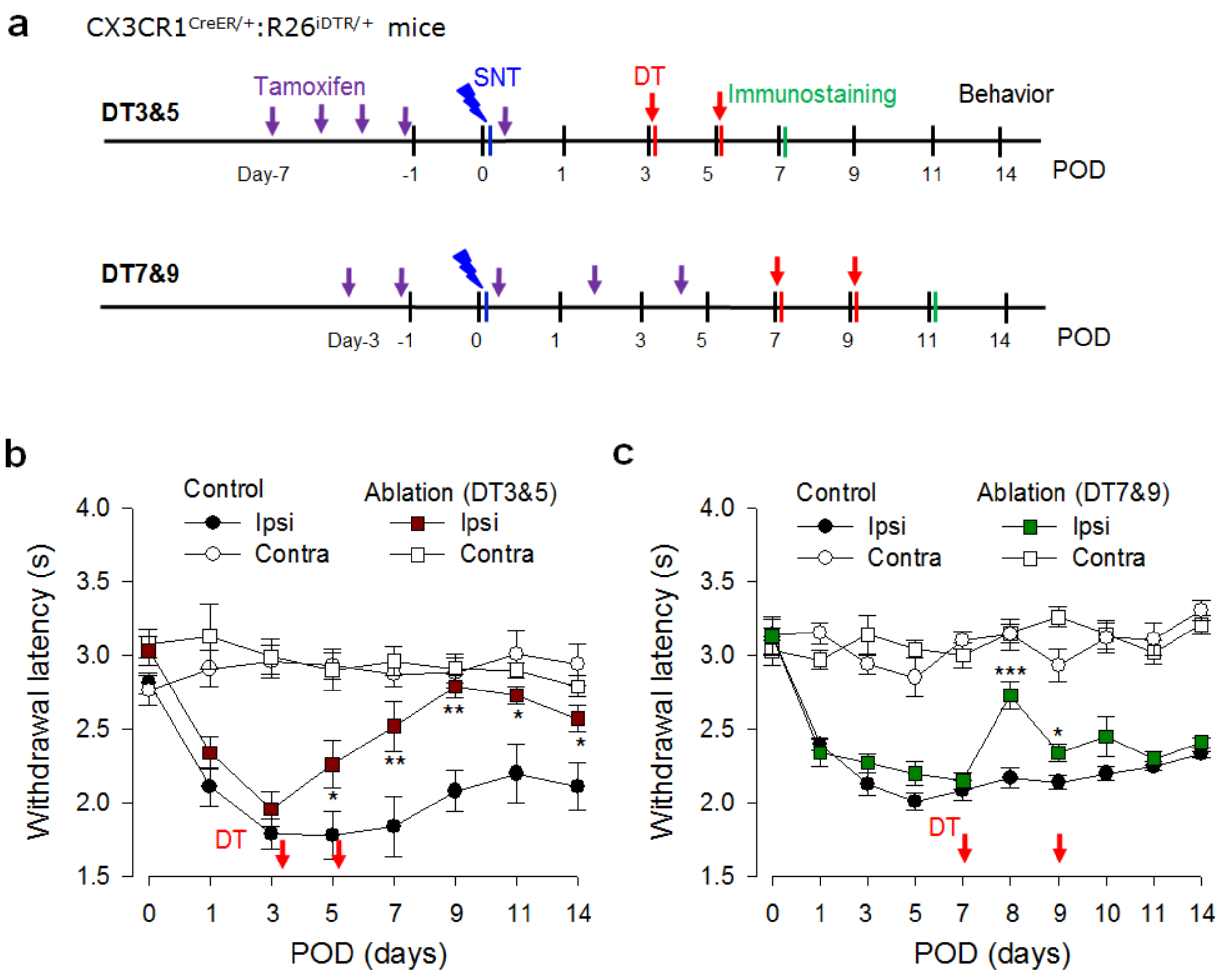

Supplementary Figure 7. Early but not late CX_3CR1^+ cell ablation strongly reverses thermal hyperalgesia. (a) An experimental diagram showing the timeline of drug treatments, SNT surgery and behavioral test in mice treated with DT at POD3 and 5 after SNT (DT3&5) or at POD7 and 9 after SNT (DT7&9). **(b)** Thermal hyperalgesia were partially reversed in DT3&5 compared with control group (n= 12 for ablation group, n = 7 for control group). **(c)** Thermal hyperalgesia in the DT7&9 group were transiently attenuated at 24 h after first DT treatment and returned to comparable level of control at POD10 and later (n = 9 for ablation, n = 7 for control). *$p < 0.05$, **$p < 0.01$, t-test. Data were obtained from $CX_3CR1^{CreER/+}$:$R26^{iDTR/+}$ mice with DT (control) and TM + DT (ablation) groups.

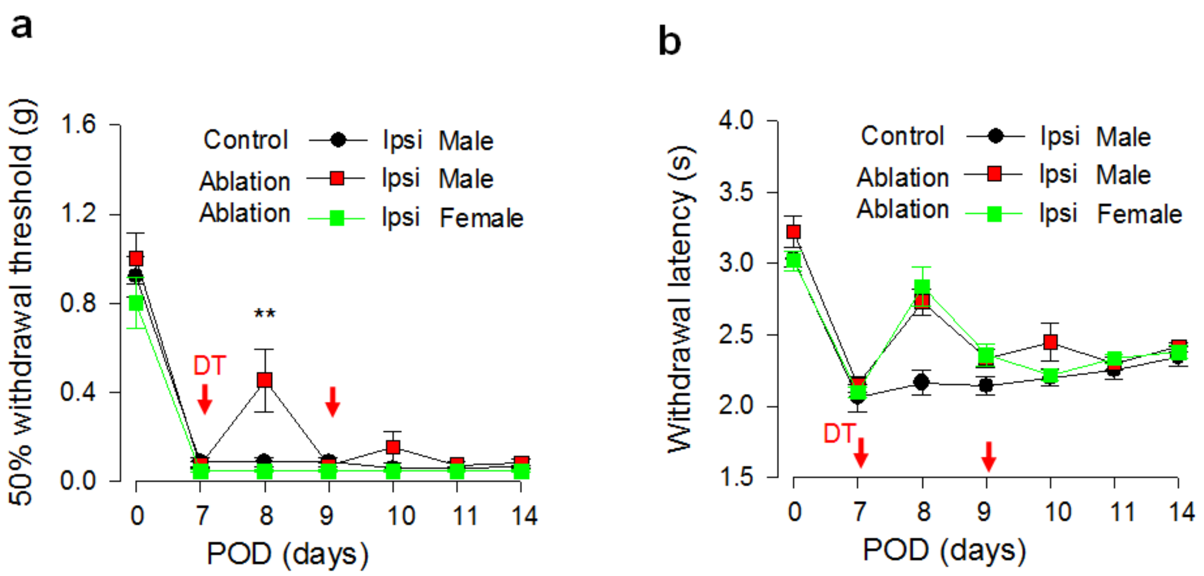

Supplementary Figure 8. Late CX_3CR1^+ cell ablation does not affect the mechanical allodynia but transiently attenuates thermal hyperalgesia in female mice. The female mice received the same DT treatment at POD7 and POD9 as the male mice did. Behavior measurement showing that (**a**) mechanical allodynia was not affected at any time point, while (**b**) thermal hyperalgesia was transiently attenuated in the DT treated female mice (n = 9 for male ablation group, n = 8 for female ablation group and n = 7 for control groups, $^{**}p < 0.01$, U-test, male vs. female for ablation groups). $CX_3CR1^{CreER/+}$:$R26^{iDTR/+}$ male and female mice with DT only were considered to be controls and mice with both TM + DT treatment was ablation group.

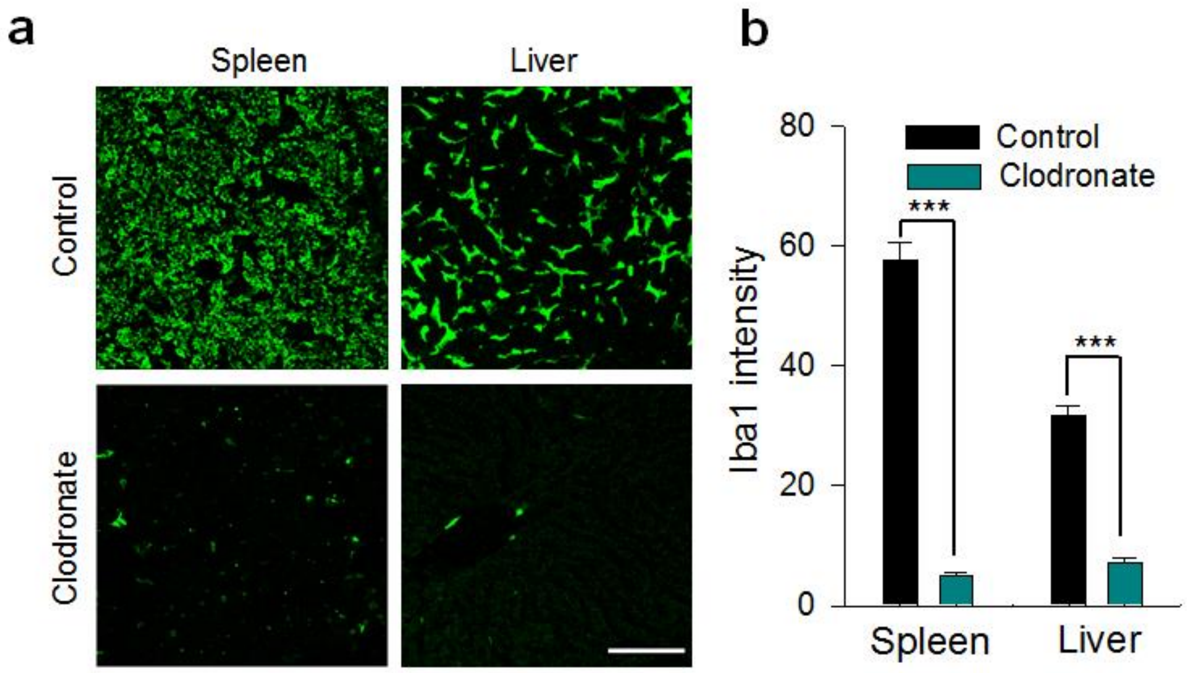

Supplementary Figure 9. Macrophage depletion in spleen and liver with clodronate treatment. (a) Representative images of Iba1 staining show macrophage depletion in both spleen and liver. Scale bar, 50μm. **(b)** Statistical analysis of the total fluorescent intensities of Iba1 signals in the spleen and liver of control and clodronate-treated groups (n = 3 for each group, ***$p < 0.001$, t-test).

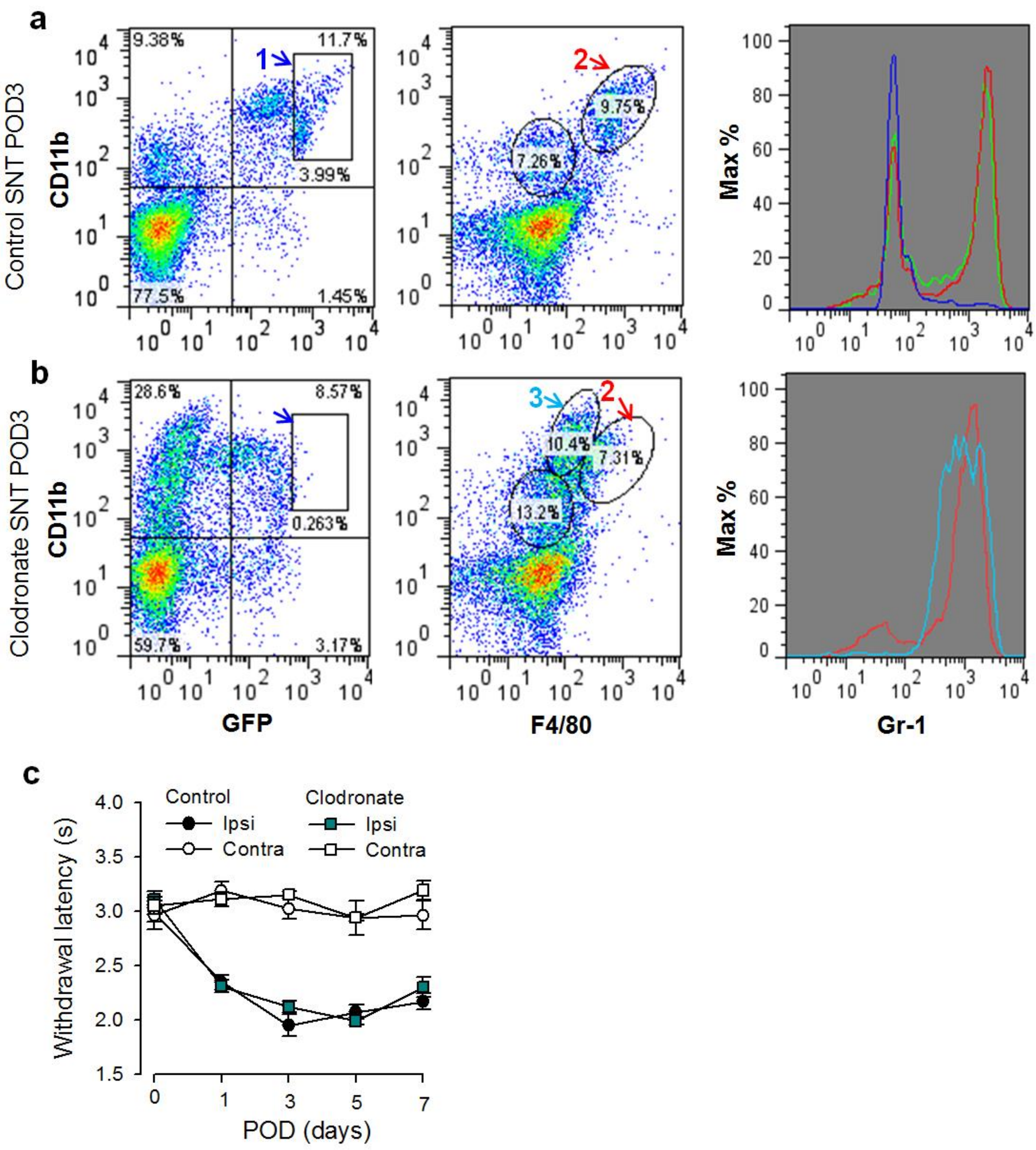

Supplementary Figure 10. Depletion of blood monocytes by clodronate treatment. (a) Flow cytometry of blood monocytes from non-clodronate treated $CX_3CR1^{GFP/+}$ mice. Left: monocytes from a SNT POD3 $CX_3CR1^{GFP/+}$ mouse showing non-altered $CD11b^+$ CX_3CR1^{low} and $CD11b^+$ CX_3CR1^{high} (box 1) populations; Middle: F4/80 signals from the SNT POD3 mice showing that both

CX_3CR1^{low} and CX_3CR1^{high} population were F4/80^{high} (circle 2). Right: Gr-1 signal distribution from the $CD11b^+$F4/80^{high} population showing the similar two separated Gr-1 expressing populations in both the naïve (green) and the SNT POD3 (red) mice. The blue line: the Gr-1 signal from the $CD11b^+$ CX_3CR1^{high} (box1) population showing that almost all $CD11b^+$ CX_3CR1^{high} were Gr-1^{low}. **(b)** The blood monocytes cytometry from a clodronate treated SNT POD3 $CX_3CR1^{GFP/+}$mice. Left: CD11b and Cx3cr1 signals showing complete depletion of the $CD11b^+$ CX_3CR1^{high} population (box 1), but a dramatic increase in $CD11b^+$ CX_3CR1^- population; Middle: The F4/80 signals showing a lower F4/80 expression in the altered $CD11b^+$ population after clodronate treatment; Right: Gr-1 signals from the remained $CD11b^+$ populations (red: from circle 2 and light blue: from circle 3) showing the absence of Gr-1^{low} population after clodronate treatment. Data were repeated from 3 animals for each group. **(c)** Monocyte depletion with clodronate prior to SNT surgery in WT mice did not affect thermal hyperalgesia development (n = 6 for clodronate group, n = 7 for control group, $p > 0.05$, control vs. clodronate t-test). The data were obtained from C57BL/6 mice with or without clodronate treatment.

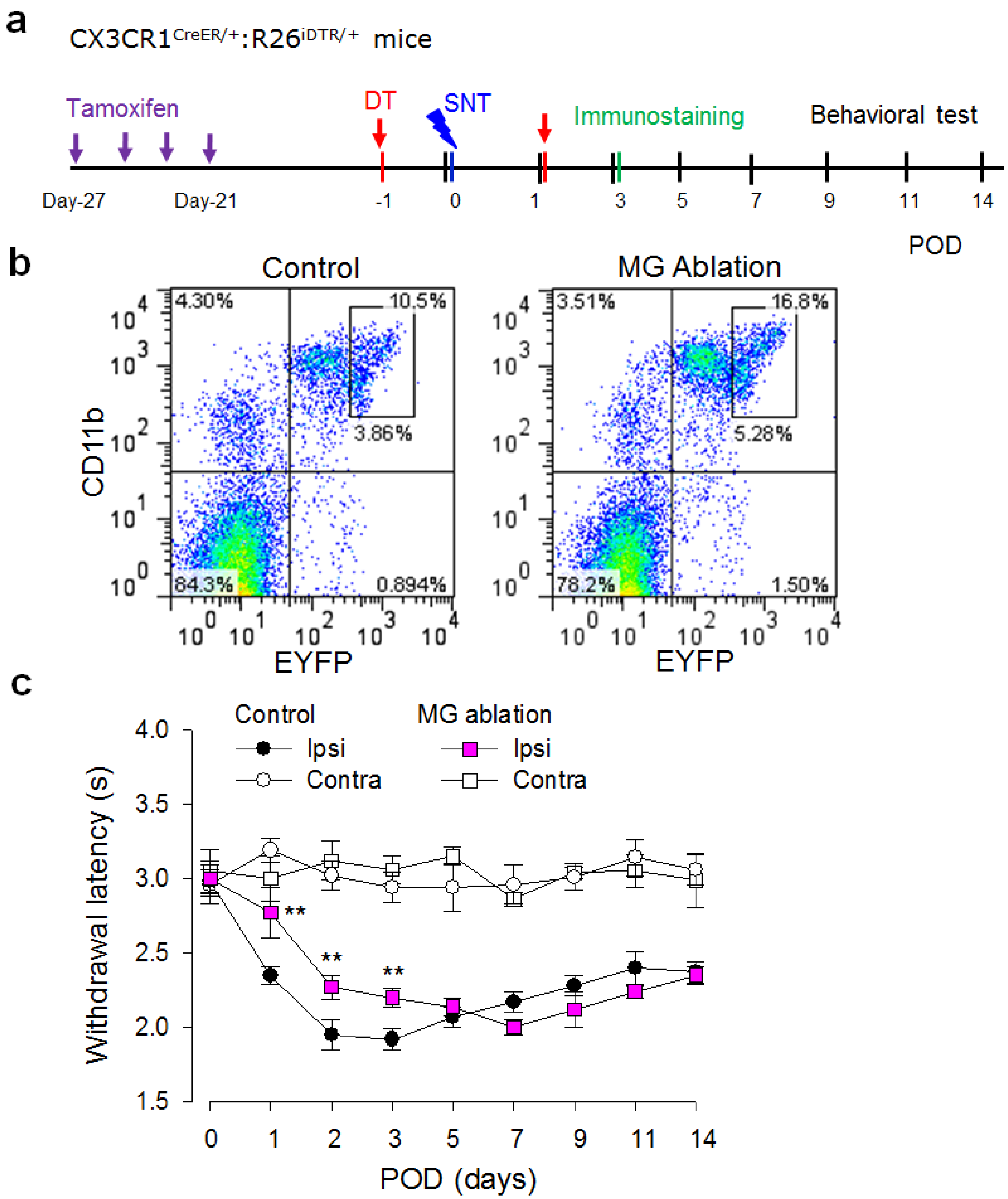

Supplementary Figure 11. The microglia ablation delayed but did not prevent the development of thermal hyperalgesia. (a) An experimental diagram showing the timeline of drug treatments, SNT surgery and behavioral tests. **(b)** Blood monocytes cytometry showing that the blood CD11b$^+$CX$_3$CR1$^+$ population was not affected by DT treatment with 3-week interval prior to the TM

treatment. **(c)** Behavior measurement showing that thermal hyperalgesia was partially reversed at POD1-3 in the microglia (MG) ablation group and was returned to levels comparable to the control group at POD5-14. (n = 11 for MG ablation group, n = 8 for control, $^{**}p < 0.01$, MG ablation ipsi vs. control ipsi, t-test). Data were obtained from $CX_3CR1^{CreER/+}$:$R26^{iDTR/+}$ mice with DT (control) and TM + DT (MG ablation) groups.

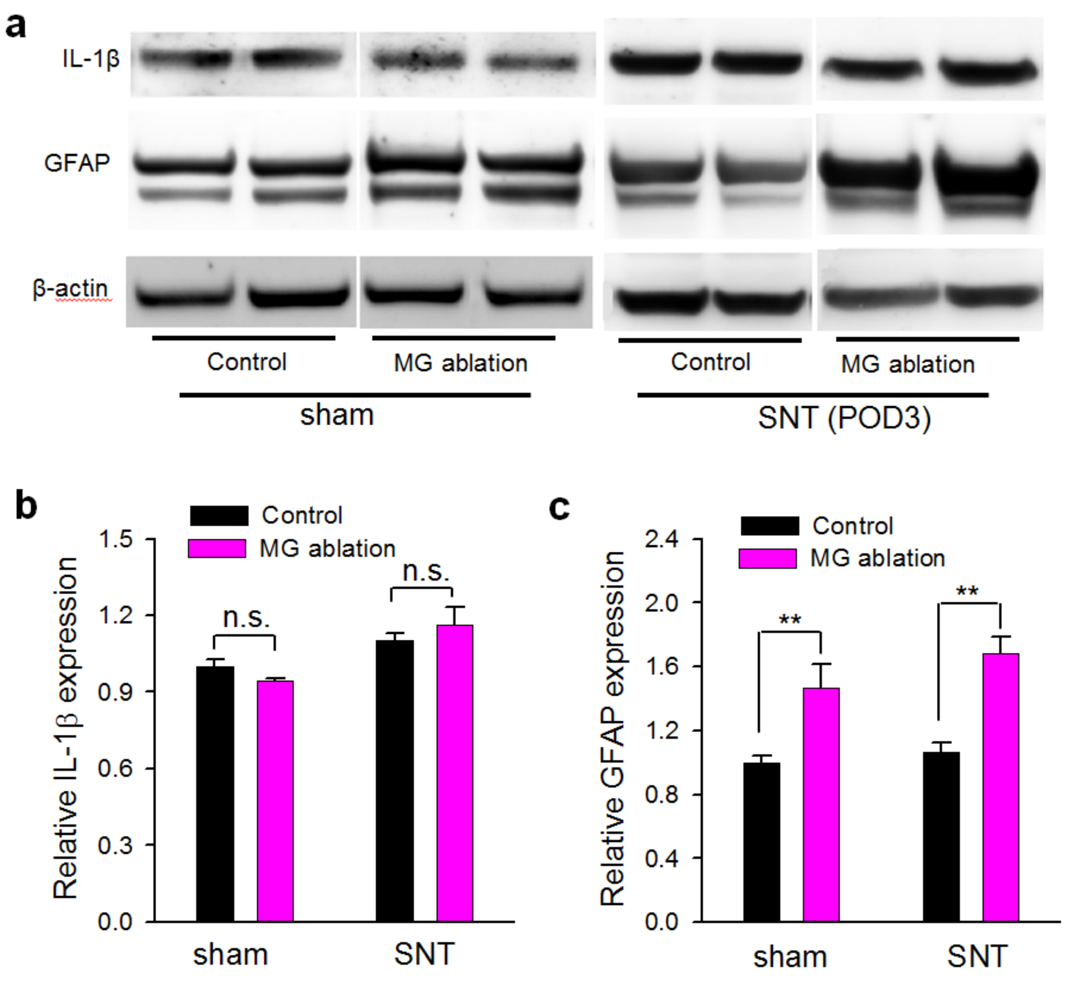

Supplementary Figure 12. IL-1β and GFAP expression about microglia ablation. (**a**) Western blot from POD3 spinal cord samples of SNT and sham groups showing similar cytokine IL-1β up-regulation in the control and microglia ablation groups. However, there is increased GFAP expression in microglia ablation mice compared with those in control mice in both sham and SNT groups. Full uncropped blots are shown in Supplementary Figure 14. (**b**) Quantification data showing that cytokine IL-1β expression was not altered in the central microglia ablation mice. (**c**) Quantification data showing that GFAP expression was increased in both sham and SNT groups after microglia ablation. (n = 4 for sham groups, respectively, n = 5 for the SNT groups, respectively). ***p* < 0.01, t-test. Data were obtained from $CX_3CR1^{CreER/+}$:$R26^{iDTR/+}$ mice with DT (control) and TM + DT (MG ablation) groups.

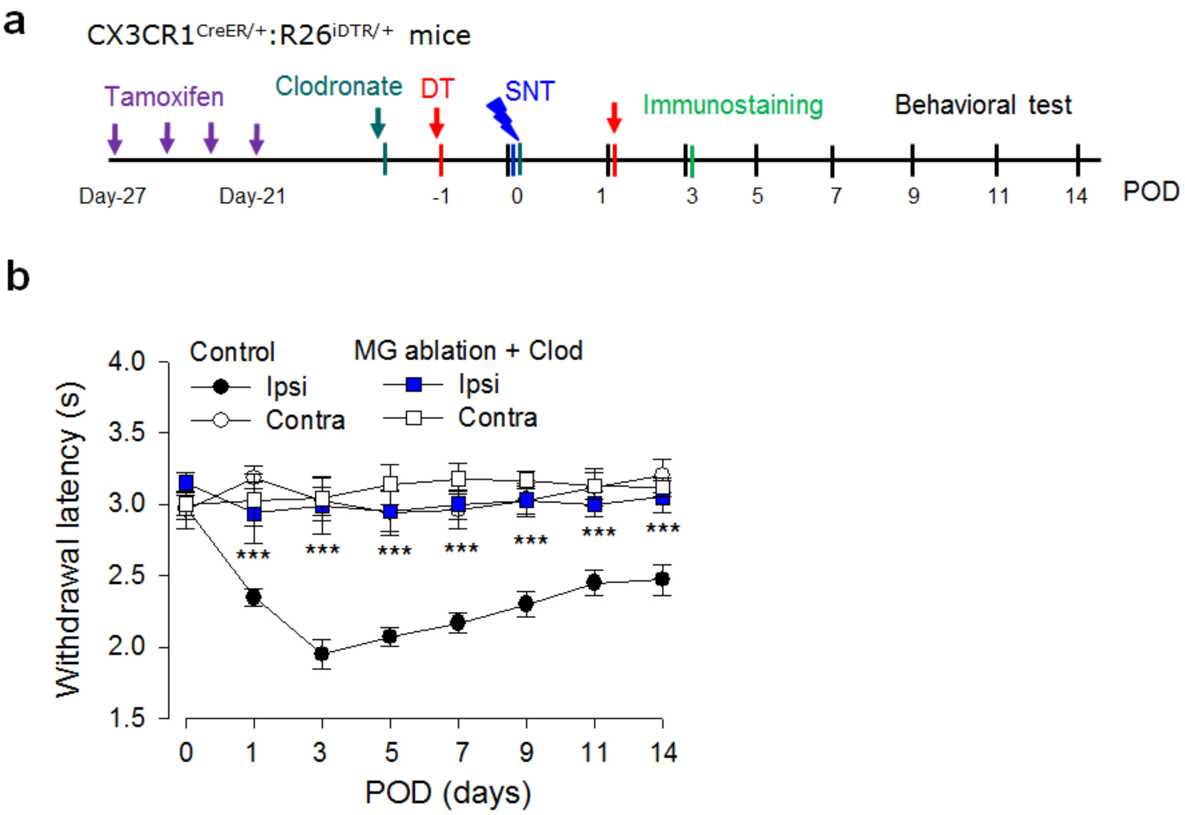

Supplementary Figure 13. Microglia ablation plus peripheral monocyte depletion prevent thermal hyperalgesia development. (a) An experimental diagram showing the timeline of drug treatments, SNT surgery and behavioral tests. **(b)** Behavioral measurement showing that thermal hyperalgesia was totally prevented in mice with microglia ablation plus monocyte depletion (n = 8 for ablation, n = 7 for control group. ***$p < 0.001$, U-test). Data were obtained from $CX_3CR1^{CreER/+}$:$R26^{iDTR/+}$ mice with DT (control) and TM + DT+ clodronate (ablation) groups.

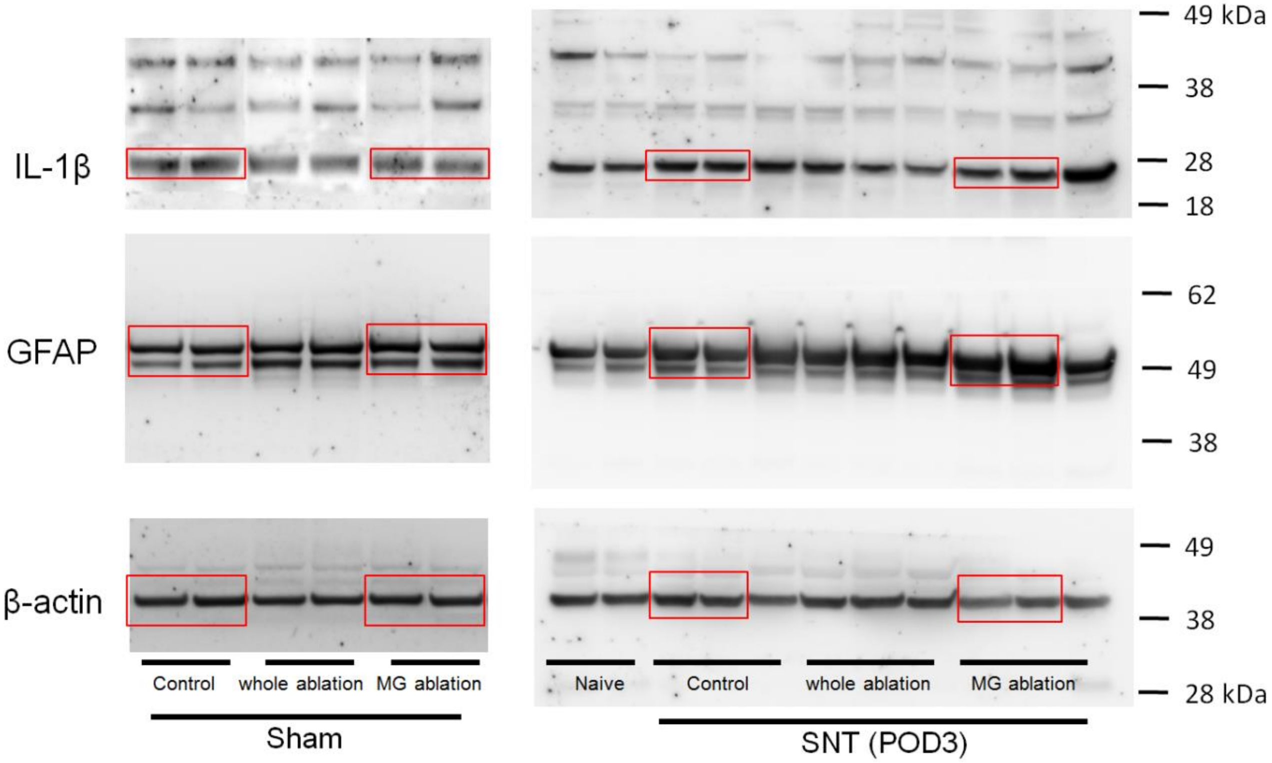

Supplementary Figure 14. Full uncropped Western blot images corresponding to Supplementary Figure 12. The red boxes indicated the blots shown in Supplementary Figure 12. n= 4 for sham groups, n = 5 for SNT groups, respectively.
